# Synthesis and biological evaluation of pyrrolidine-based T-type calcium channel inhibitors for the treatment of neuropathic pain

**DOI:** 10.1080/14756366.2018.1513926

**Published:** 2018-09-20

**Authors:** Hak Kyun Yang, Woo Seung Son, Keon Seung Lim, Gun Hee Kim, Eun Jeong Lim, Changdev G. Gadhe, Jae Yeol Lee, Kyu-Sung Jeong, Sang Min Lim, Ae Nim Pae

**Affiliations:** a Convergence Research Center for Diagnosis, Treatment and Care System of Dementia, Korea Institute of Science and Technology, Seoul, Republic of Korea;; b Department of Chemistry, Yonsei University, Seoul, Republic of Korea;; c 1ST Biotherapeutics Inc., Seongnam, Gyeonggi-do, Republic of Korea;; d Research Institute for Basic Sciences and Department of Chemistry, College of Sciences, Kyung Hee University, Seoul, Republic of Korea;; e Division of Bio-Medical Science and Technology, Korea University of Science and Technology, Daejon, Republic of Korea;; f Division of Bio-Medical Science & Technology, KIST School, Korea University of Science and Technology, Seoul, Republic of Korea

**Keywords:** Neuropathic pain, T-type calcium channel, pyrrolidine, spinal nerve ligation, streptozotocin

## Abstract

The treatment of neuropathic pain is one of the urgent unmet medical needs and T-type calcium channels are promising therapeutic targets for neuropathic pain. Several potent T-type channel inhibitors showed promising *in vivo* efficacy in neuropathic pain animal models and are being investigated in clinical trials. Herein we report development of novel pyrrolidine-based T-type calcium channel inhibitors by pharmacophore mapping and structural hybridisation followed by evaluation of their Ca_v_3.1 and Ca_v_3.2 channel inhibitory activities. Among potent inhibitors against both Ca_v_3.1 and Ca_v_3.2 channels, a promising compound **20n** based on *in vitro* ADME properties displayed satisfactory plasma and brain exposure in rats according to *in vivo* pharmacokinetic studies. We further demonstrated that **20n** effectively improved the symptoms of neuropathic pain in both SNL and STZ neuropathic pain animal models, suggesting modulation of T-type calcium channels can be a promising therapeutic strategy for the treatment of neuropathic pain.

## Introduction

Neuropathic pain is chronic pain originated from lesions or diseases of the peripheral or central somatosensory nervous system^1^. It is usually associated with unpleasant feeling such as a burning, abnormal crawling, or electric shock-like sensations and is further characterised by evoked pain after stimuli such as hypersensitivity and summation. Current therapeutic options for neuropathic pain include anti-convulsants such as sodium and calcium channel blockers, anti-depressants, opioids, NSAIDs, cannabinoids, topical agents, and combinations of these drugs^2^^,^^3^. However, administration of these drugs only reduce 30–50% of pain in approximately 50% of neuropathic pain patients^4^^,^^5^. This low efficacy issue can be attributed to the substantial heterogeneity of pain mechanisms, and requests precise elucidation of underlying mechanisms for different types of neuropathic pain in order to improve both diagnosis and prescription. Thus, identification of potential biomarkers for each type of neuropathic pain and subsequent development of chemical probes that can modulate the biomarkers will provide exciting opportunities to better understand the heterogeneity of neuropathic pain and produce efficacious pain drugs^6^^,^^7^.

One of the encouraging strategies to treat neuropathic pain is modulation of intracellular calcium levels^8^^,^^9^ either directly by controlling voltage-gated calcium channels or indirectly by regulating receptors such as nicotinic acetylcholine receptors^10^^,^^11^. Among several voltage-gated calcium channels, T-type calcium channels have received great attention as one of the promising molecular targets for neuropathic pain^12^^,^^13^. These are low voltage-activated (LVA) channels composed of α1 subunit single pore, and three isoforms of T-type calcium channels have been identified^14^: Ca_v_3.1 (α_1G_)^15^, Ca_v_3.2 (α_1H_)^16^, Ca_v_3.3 (α_1I_)^17^. In contrast to high voltage-activated (HVA) channels, T-type calcium channels are activated even after a slight depolarisation of the cell membrane, functioning at near-resting membrane potentials^18^. Due to this unique voltage sensitivity, T-type calcium channels can regulate neuronal excitability and oscillatory behaviour^19^.

Among T-type calcium channels, Ca_v_3.2 is the major isoform expressed in primary sensory afferent neurons and spinal cord^20^, and it has been strongly implicated that Ca_v_3.2 channel contributes to the development of neuropathic pain^12^. For example, in a neuropathic pain rat model derived by a chronic constriction injury (CCI), the expression of Ca_v_3.2 channel is highly up-regulated, and delivery of Ca_v_3.2-specific antisense oligonucleotide generated robust pain-relieving effects^20^. Furthermore, in a STZ-induced neuropathic pain model, the level of Ca_v_3.2 expression is also increased and specific *in vivo* knock-down of Ca_v_3.2 effectively alleviated mechanical and thermal hypersensitivity^21^. In addition, Ca_v_3.1 channels are mainly expressed in dorsal horn neurons^22^, and its significance in neuropathic pain is also well reported. It was demonstrated that after spinal nerve ligation (SNL) Ca_V_3.1 knock-out mice showed a higher threshold to mechanical stimuli than their wild-type litter mates, and thermal hypersensitivity was also substantially decreased^23^. Furthermore, the mechanical hypersensitivity was markedly attenuated after induction of trigeminal neuropathy in the Ca_V_3.1 knock-out mice as compared to wild type mice^24^.

Based on these observations, it was believed that T-type calcium channel inhibitors would provide effective treatment options for neuropathic pain^13^, leading to development of a variety of T-type calcium channel blockers, such as Mibefradil^25^, ethosuximide^26^, and (3β,5α,17β)-17-hydroxyestrane-3-carbonitrile (ECN)^27^. In fact, treatment of Mibefradil or ethosuximide in the rat CCI model relieved behavioural symptoms of neuropathic pain^28^. It was shown that ECN also alleviated mechanical and thermal hypersensitivity in rats with neuropathic pain^29^ and in diabetic ob/ob mice^30^. Although these early T-type channel inhibitors showed great promise for the treatment of neuropathic pain, however, they suffered relatively low selectivity against other ion channels, especially voltage-gated sodium channels in neurons raising adverse side effect issues^25^^,^^31^^,^^32^. Moreover, Mibefradil was withdrawn just 1 year after the FDA approval due to drug–drug interactions^33^.

Thus, development of selective T-type channel inhibitors is highly desired to treat neuropathic pain with minimal side effects. Recently, selective T-type channel inhibitors such as R-(-)-efonidipine^34^, TTA-P2^35^, ML-218^36^, ABT-639^37^, Z-944^38^, and benzodiazepine- and dihydropyrazole-based Actelion inhibitors^39^^,^^40^ have been developed, and some of them presented robust analgesic effects in several neuropathic pain models^41^^,^^42^, illustrating selective T-type calcium channel inhibitors hold strong potential to be effective treatment options for neuropathic pain.

Herein we describe our ongoing efforts to develop selective T-type channel inhibitors, identifying pyrrolidine derivatives as potent T-type channel inhibitors after evaluation of Ca_v_3.1 (α_1G_) and Ca_v_3.2 (α_1H_) T-type calcium channel blocking activities with fluorescence-based FDSS6000 assay^43^ and whole-cell patch clamp recordings^44^. Pharmacokinetic parameters of a promising compound **20n** and its *in vivo* analgesic efficacy in two different neuropathic pain models are investigated.

## Materials and methods

### Chemistry

Commercially available reagents and solvents were used without further purification. Air- and moisture-sensitive reactions were performed under a positive pressure of nitrogen. All reactions were monitored by analytical thin layer chromatography (TLC) using glass pates pre-coated with silica gel from Merck (0.25 mm, 60 Å pore-size) impregnated with a fluorescent indicator (254 nm). Visualisation of TLC was carried out by ultraviolet light (λ = 254 and 365 nm). Flash column chromatography was performed on Merck silica gel 60 (70–230 mesh). ^1^H NMR were recorded on Bruker AVANCE II 400 (400 MHz), 300 (300 MHz) NMR spectrometers at 23 °C. Proton chemical shifts are expressed in parts per million (ppm, δ scale) and are referenced to residual protium in the NMR solvent (CDCl_3_, δ 7.26; DMSO-d_6_, δ 2.50). Peak splitting patterns are presented as follows: br = broad, s = singlet, d = doublet, t = triplet, q = quartet, m = multiplet, dd = doublet of doublet. Coupling constants (*J*) are recorded in hertz (Hz) values. 13C NMR spectra were recorded on Bruker AVANCE II 400 (100 MHz), 300 (75 MHz) NMR spectrometers at 23 °C. Carbon chemical shifts are expressed in parts per million (ppm, δ scale) and are referenced to the carbon resonances of the NMR solvent (CDCl_3_, δ 77.0; DMSO-d_6_, δ 39.50). High-resolution mass spectra (HRMS) were recorded on a LTQ Orbitrap (Thermo Electron Corporation). Purity was measured using Waters e2695/2489 HPLC instrument (1 ml/min flow rate; H_2_O/CH_3_CN 90:10 → 0:100 in 15 min; 10 μL of injection volume; λ = 254, 280 nm).

### Synthesis of (S)-5-isobutyl-1-phenyl-N-(pyrrolidin-3-ylmethyl)-1H-pyrazole-3-carboxamide (15)

(*R*)-*tert*-Butyl 3-((5-isobutyl-1-phenyl-1*H*-pyrazole-3-carboxamido)methyl)pyrrolidine-1-carboxylate **14** (2.93 g, 6.87 mmol) in anhydrous DCM (15 ml) was treated with trifluoroacetic acid (10 ml) and stirred at room temperature for 2 h. The reaction mixture was evaporated and neutralised by adding an aqueous solution of saturated sodium bicarbonate. The aqueous layer was extracted with DCM, dried over sodium sulfate and concentrated *in vacuo*. The desired compound (2.22 g) was obtained. Yield 99%; ^1^H NMR (400 MHz, CDCl_3_) *δ*: 7.54–7.40 (m, 5H), 6.75 (s, 1H), 3.45 (d, *J* = 7.2 Hz, 2H), 3.30–3.24 (br, 2H), 3.17–3.14 (m, 1H), 3.00 (dd, *J* = 11.6, 6.4 Hz, 1H), 2.63–2.56 (m, 1H), 2.51 (d, *J* = 7.2 Hz, 2H), 2.10–2.05 (m, 1H), 1.88–1.81 (m, 1H), 1.73–1.68 (m, 1H), 0.87 (d, *J* = 6.8 Hz, 6H); 13C NMR (100 MHz, CDCl_3_) *δ*: 162.89, 146.04, 145.22, 139.16, 129.27, 128.84, 125.84, 106.26, 48.29, 44.70, 40.67, 38.65, 34.93, 28.19, 22.20; HPLC purity 100%; HR-MS *m/z* [M + H]^+^ (ESI^+^) calcd. for C_19_H_27_N_4_O = 327.2179, found 327.2180.

### Synthesis of (R)-N-((1-(3,3-dimethylbutyl)pyrrolidin-3-yl)methyl)-5-isobutyl-1-phenyl-1H-pyrazole-3-carboxamide (16)

(*S*)-1-methyl-3-propyl-*N*-(pyrrolidin-3-ylmethyl)-1*H*-pyrazole-5-carboxamide **15** (100 mg, 0.40 mmol) and 3,3-dimethylbutanal (50 μL, 0.40 mmol) were dissolved in anhydrous DCM. Molecular sieves were added to the mixture. The mixture was stirred at room temperature for 2 h. NaBH(OAc)_3_ (254 mg, 1.20 mmol) was added to the mixture. The reaction was stirred at room temperature for 10 h. It was quenched with an aqueous solution of saturated sodium bicarbonate, water and extracted with DCM. The residue was dried over magnesium sulfate and concentrated *in vacuo*. The residue was purified via column chromatography on silica gel (DCM/MeOH =10:1) to obtain the desired compound (16 mg). Yield: 12%; ^1^H NMR (300 MHz, CDCl_3_) *δ*: 7.52–7.47 (m, 3H) 7.43–7.40 (m, 2H) 6.75 (s, 1H) 3.50–3.44 (m, 2H) 2.80–2.52 (m, 7H) 2.51 (d, *J* = 7.2 Hz, 2H) 2.14 (m, 1H) 1.84 (m, 1H) 1.69 (m, 1H) 1.51 (m, 2H) 0.89 (s, 9H) 0.86 (d, *J* = 6.78 Hz, 6H); 13C NMR (75 MHz, CDCl_3_) *δ*: 162.91, 146.15, 145.29, 139.27, 129.32, 128.83, 125.90, 106.51, 57.09, 53.21, 52.54, 41.66, 39.25, 37.78, 35.09, 29.76, 29.18, 28.32, 27.58, 22.32.

### General procedure for the preparation of compounds 20a-p

The pyrrolidine **15** and potassium carbonate in acetonitrile was stirred at room temperature for 30 min. The tosylate **19a–p** in acetonitrile was added dropwise and refluxed for 24 h. The reaction mixture was cooled to room temperature, quenched with water and extracted with ethyl acetate. It was dried over sodium sulfate and concentrated *in vacuo*. The residue was purified via column chromatography on silica gel, and the desired compound was obtained.

#### (R)-5-Isobutyl-1-phenyl-N-((1-(4-(trifluoromethyl)phenethyl)pyrrolidin-3-yl)meth-yl)-1H-pyrazole-3-carboxamide (20a)

Yield 72%; ^1^H NMR (300 MHz, CDCl_3_) *δ*: 7.52–7.40 (m, 8H) 7.28–7.24 (m, 2H) 6.78 (s, 1H) 3.48–3.43 (m, 2H) 2.88–2.83 (m, 2H) 2.76–2.65 (m, 4H) 2.56–2.47 (m, 5H) 2.08–2.02 (m, 1H) 1.90–1.81 (m, 1H) 1.63–1.57 (m, 1H) 0.88 (d, *J* = 6.60 Hz, 6H); 13C NMR (75 MHz, CDCl_3_) *δ*: 162.98 146.09 145.37 141.22 139.26 129.60 129.34 129.11 128.88 125.90 125.77 125.67 122.27 106.4857.15 56.65 53.53 41.65 37.93 35.08 32.60 28.32 27.61 22.32.

#### (R)-5-Isobutyl-1-phenyl-N-((1-(3-(trifluoromethyl)phenethyl)pyrrolidin-3-yl)methy-l)-1H-pyrazole-3-carboxamide (20b)

Yield 35%; ^1^H NMR (300 MHz, CDCl_3_) *δ*: 7.51–7.30 (m, 9H), 6.79 (s, 1H), 3.47–3.38 (m, 2H), 2.87–2.82 (m, 2H), 2.78–2.65 (m, 4H), 2.58–2.49 (m, 5H), 2.08 (s, 1H), 1.9–1.78 (m, 1H), 1.64–1.53 (m, 1H), 0.89 (d, *J* = 6.6 Hz, 6H); 13C NMR (75 MHz, CDCl_3_) *δ* 162.55 146.85 145.07 141.24 139.45 132.08 130.74 130.43 129.25 128.72 128.70 125.96 125.57 125.31 125.28 122.91 122.88 58.38 57.54 53.82 43.90 37.06 35.18 35.12 28.37 28.32 22.34.

#### (R)-5-Isobutyl-1-phenyl-N-((1-(2-(trifluoromethyl)phenethyl)pyrrolidin-3-yl)methy-l)-1H-pyrazole-3-carboxamide (20c)

Yield 27%; ^1^H NMR (300 MHz, CDCl_3_) *δ*: 7.58–7.24 (m, 9H), 6.75 (s, 1H), 3.46–3.43 (m, 2H), 2.97–2.94 (m, 2H), 2.78–2.46 (m, 9H), 2.18–1.99 (m, 1H), 1.88–1.78 (m, 1H), 1.63–1.55 (m, 1H), 0.87 (d, *J* = 6.6 Hz, 6H); 13C NMR (75 MHz, CDCl_3_) *δ*: 162.54 146.82 145.08 139.43 138.89 131.72 131.59 129.27 128.80 128.69 128.41 126.37 126.13 125.97 125.89 125.82 125.74 122.74 106.53 58.24 57.74 53.68 43.85 37.14 35.12 32.10 28.41 28.33 22.35.

#### (R)-N-((1-(3-fluorophenethyl)pyrrolidin-3-yl)methyl)-5-isobutyl-1-phenyl-1H-pyrazole-3-carboxamide (20d)

Yield 70%; ^1^H NMR (400 MHz, CDCl_3_) *δ*: 7.49–7.38 (m, 6H), 7.20–7.15 (m, 1H), 6.90–6.81 (m, 3H), 6.76 (s, 1H), 3.47–3.39 (m, 2H), 2.79–2.61 (m, 6H), 2.54–2.45 (m, 5H), 2.04–1.98 (m, 1H), 1.86–1.79 (m, 1H), 1.61–1.54 (m, 1H), 0.85 (d, *J* = 6.6 Hz, 6H); 13C NMR (100 MHz, CDCl_3_) *δ*: 162.78 (d, *J*_C-F_ = 243.7 Hz), 162.50, 146.86, 144.97, 142.97 (d, *J*_C-F_ = 7.2 Hz), 139.43, 129.63 (d, *J*_C-F_ = 8.2 Hz), 129.21, 128.63, 125.88, 124.23 (d, *J*_C-F_ = 2.7 Hz), 115.39 (d, *J*_C-F_ = 20.8 Hz), 112.76 (d, *J*_C-F_ = 20.9 Hz), 106.50, 58.34, 57.49, 53.82, 43.92, 37.01, 35.28, 35.07, 28.34, 28.26, 22.29; HR-MS *m/z* [M + H]^+^ (ESI^+^) calcd. for C_27_H_34_FN_4_O = 449.2711, found 449.2707.

#### (R)-N-((1-(3,4-difluorophenethyl)pyrrolidin-3-yl)methyl)-5-isobutyl-1-phenyl-1H-p-yrazole-3-carboxamide (20e)

Yield 75%; ^1^H NMR (400 MHz, CDCl_3_) *δ*: 7.47–7.37 (m, 6H), 7.01–6.94 (m, 1H), 6.92–6.87 (m, 1H), 6.81–6.79 (br m, 1H), 6.75 (s, 1H), 3.43–3.38 (m, 2H), 2.73–2.58 (m, 6H), 2.51–2.43 (m, 5H), 2.04–1.95 (m, 1H), 1.85–1.78 (m, 1H), 1.59–1.51 (m, 1H), 0.84 (d, *J* = 6.6 Hz, 6H); 13C NMR (100 MHz, CDCl_3_) *δ*: 162.49, 150.05 (d, *J*_C-F_ = 245.6 Hz), 149.92 (d, *J*_C-F_ = 245.7 Hz), 148.79 (d, *J*_C-F_ = 244.1 Hz), 148.66 (d, *J*_C-F_ = 244.1 Hz), 146.84, 144.98, 139.42, 137.41 (d, *J*_C-F_ = 4.2 Hz), 137.35 (d, *J*_C-F_ = 4.0 Hz), 129.19, 128.62, 125.87, 124.39 (d, *J*_C-F_ = 3.5 Hz), 124.33 (d, *J*_C-F_ = 3.5 Hz), 117.23 (d, *J*_C-F_ = 16.7 Hz), 116.80 (d, *J*_C-F_ = 16.8 Hz), 106.47, 58.28, 57.43, 53.76, 43.88, 36.99, 35.04, 34.64, 28.29, 28.25, 22.26; HR-MS *m/z* [M + H]^+^ (ESI^+^) calcd. for C_27_H_33_F_2_N_4_O = 467.2616, found 467.2612.

#### (R)-N-((1-(3,5-difluorophenethyl)pyrrolidin-3-yl)methyl)-5-isobutyl-1-phenyl-1H-pyrazole-3-carboxamide (20f)

Yield 70%; ^1^H NMR (400 MHz, CDCl_3_) *δ*: 7.51–7.37 (m, 6H), 6.75 (s, 1H), 6.64–6.55 (m, 3H), 3.46–3.39 (m, 2H), 2.76–2.59 (m, 6H), 2.51–2.46 (m, 5H), 2.03–1.97 (m, 1H), 1.85–1.78 (m, 1H), 1.58–1.54 (m, 1H), 0.84 (d, *J* = 6.6 Hz, 6H); 13C NMR (100 MHz, CDCl_3_) *δ*: 164.17, 162.94 (d, *J*_C-F_ = 245.9 Hz), 162.81 (d, *J*_C-F_ = 246.2 Hz), 146.83, 145.03, 144.29 (d, *J*_C-F_ = 18.0 Hz), 144.29, 139.43, 129.23, 128.68, 125.91, 111.42 (d, *J*_C-F_ = 6.4 Hz), 111.24 (d, *J*_C-F_ = 6.2 Hz), 106.49, 101.51 (d, *J*_C-F_ = 25.3 Hz), 101.25 (d, *J*_C-F_ = 25.1 Hz), 58.30, 56.98, 53.75, 43.88, 37.00, 35.20, 35.07, 28.32, 28.28, 22.30; HR-MS *m/z* [M + H]^+^ (ESI^+^) calcd. for C_27_H_33_F_2_N_4_O = 467.2616, found 467.2613.

#### (R)-N-((1-(2,3-difluorophenethyl)pyrrolidin-3-yl)methyl)-5-isobutyl-1-phenyl-1H-pyrazole-3-carboxamide (20g)

Yield 76%; ^1^H NMR (400 MHz, CDCl_3_) *δ*: 7.46–7.35 (m, 6H), 6.94–6.84 (m, 3H), 6.72 (s, 1H), 3.43–3.35 (m, 2H), 2.82–2.78 (m, 2H), 2.70–2.59 (m, 4H), 2.52–2.43 (m, 5H), 2.00–1.95 (m, 1H), 1.83–1.76 (m, 1H), 1.56–1.52 (m, 1H), 0.82 (d, *J* = 6.6 Hz, 6H); 13C NMR (100 MHz, CDCl_3_) *δ*: 162.45, 150.53 (d, *J*_C-F_ = 245.7 Hz), 150.40 (d, *J*_C-F_ = 245.6 Hz), 148.98 (d, *J*_C-F_ = 244.5 Hz), 148.86 (d, *J*_C-F_ = 244.6 Hz), 146.82, 139.40, 129.77, 129.64, 129.15, 128.56, 125.83, 125.44 (d, *J*_C-F_ = 3.6 Hz), 125.40 (d, *J*_C-F_ = 3.5 Hz), 123.70 (d, *J*_C-F_ = 6.8 Hz), 123.66 (d, *J*_C-F_ = 6.9 Hz), 114.84 (d, *J*_C-F_ = 16.9 Hz), 106.45, 58.20, 55.94, 53.68, 43.85, 37.04, 35.03, 28.50, 28.30, 28.23, 22.24; HR-MS *m/z* [M + H]^+^ (ESI^+^) calcd. for C_27_H_33_F_2_N_4_O = 467.2616, found 467.2609.

#### (R)-N-((1-(3-chloro-5-fluorophenethyl)pyrrolidin-3-yl)methyl)-5-isobutyl-1-phenyl-1H-pyrazole-3-carboxamide (20h)

Yield 71%; ^1^H NMR (400 MHz, CDCl_3_) *δ*: 7.55 (t, *J* = 5.5 Hz, 1H), 7.50–7.39 (m, 5H), 6.90–6.87 (m, 2H), 6.77 (s, 1H), 6.73 (d, *J* = 9.2 Hz, 1H), 3.49–3.37 (m, 2H), 2.76–2.60 (m, 6H), 2.52–2.46 (m, 5H), 2.06–1.98 (m, 1H), 1.89–1.79 (m, 1H), 1.62–1.54 (m, 1H), 0.86 (d, *J* = 6.6 Hz, 6H); 13C NMR (100 MHz, CDCl_3_) *δ*: 162.56 (d, *J*_C-F_ = 247.2 Hz), 162.51, 146.88, 144.99, 144.21 (d, *J*_C-F_ = 8.2 Hz), 139.45, 134.53 (d, *J*_C-F_ = 10.8 Hz), 129.21, 128.66, 125.90, 124.62 (d, *J*_C-F_ = 2.8 Hz), 113.99 (d, *J*_C-F_ = 21.2 Hz), 113.76 (d, *J*_C-F_ = 25.3 Hz), 106.48, 58.38, 57.00, 53.76, 43.96, 36.96, 35.08, 28.34, 28.28, 22.29; HR-MS *m/z* [M + H]^+^ (ESI^+^) calcd. for C_27_H_33_ClFN_4_O = 483.2321, found 483.2316.

#### (R)-N-((1-(4-chloro-3-fluorophenethyl)pyrrolidin-3-yl)methyl)-5-isobutyl-1-phenyl-1H-pyrazole-3-carboxamide (20i)

Yield 77%; ^1^H NMR (400 MHz, CDCl_3_) *δ*: 7.50 (t, *J* = 5.5 Hz, 1H), 7.45–7.35 (m, 5H), 7.18 (t, *J* = 7.9 Hz, 1H), 6.85 (dd, *J* = 10.1, 1.7 Hz, 1H), 6.79 (d, *J* = 8.0 Hz, 1H), 6.73 (s, 1H), 3.43–3.34 (m, 2H), 2.72–2.56 (m, 6H), 2.48–2.42 (m, 5H), 2.01–1.92 (m, 1H), 1.83–1.76 (m, 1H), 1.57–1.49 (m, 1H), 0.82 (d, *J* = 6.6 Hz, 6H); 13C NMR (100 MHz, CDCl_3_) *δ*: 162.47, 157.76 (d, *J*_C-F_ = 246.7 Hz), 146.83, 144.93, 141.50, 141.44, 139.41, 130.12, 129.17, 128.60, 125.83, 125.04 (d, *J*_C-F_ = 3.3 Hz), 118.08 (d, *J*_C-F_ = 17.5 Hz), 116.63 (d, *J*_C-F_ = 20.4 Hz), 106.46, 58.27, 57.14, 53.74, 43.89, 36.96, 35.03, 34.77, 28.28, 28.23, 22.25; HR-MS *m/z* [M + H]^+^ (ESI^+^) calcd. for C_27_H_33_ClFN_4_O = 483.2321, found 483.2316.

#### (R)-N-((1-(3,4-dichlorophenethyl)pyrrolidin-3-yl)methyl)-5-isobutyl-1-phenyl-1H-pyrazole-3-carboxamide (20j)

Yield 77%; ^1^H NMR (400 MHz, CDCl_3_) *δ*: 7.50 (t, *J* = 5.5 Hz, 1H) 7.47–7.36 (m, 5H), 7.25 (d, *J* = 8.2 Hz, 1H), 7.17 (d, *J* = 1.9. Hz, 1H), 6.92 (dd, *J* = 8.2, 2.0 Hz, 1H), 6.74 (s, 1H), 3.45–3.35 (m, 2H), 2.71–2.56 (m, 6H), 2.49–2.43 (m, 5H), 2.02–1.94 (m, 1H), 1.84–1.77 (m, 1H), 1.59–1.51 (m, 1H), 0.83 (d, *J* = 6.6 Hz, 6H); 13C NMR (100 MHz, CDCl_3_) *δ*: 162.49, 146.85, 144.95, 140.73, 139.42, 131.99, 130.47, 130.10, 129.76, 129.19, 128.62, 128.09, 125.86, 106.47, 58.31, 57.20, 53.76, 43.92, 36.95, 35.05, 34.59, 28.30, 28.25, 22.28; HR-MS *m/z* [M + H]^+^ (ESI^+^) calcd. for C_27_H_33_Cl_2_N_4_O = 499.2025, found 499.2019.

#### (R)-N-((1-(2,4-bis(trifluoromethyl)phenethyl)pyrrolidin-3-yl)methyl)-5-isobutyl-1-phenyl-1H-pyrazole-3-carboxamide (20k)

Yield 40%; ^1^H NMR (400 MHz, CDCl_3_) *δ*: 7.87 (s, 1H), 7.69 (d, *J* = 8.1 Hz, 1H), 7.52–7.40 (m, 7H), 6.79 (s, 1H), 3.53–3.41 (m, 2H), 3.04 (t, *J* = 7.9 Hz, 2H), 2.79–2.67 (m, 4H), 2.60–2.49 (m, 5H), 2.11–2.02 (m, 1H), 1.89–1.83 (m, 1H), 1.66–1.58 (m, 1H), 0.88 (d, *J* = 6.6 Hz, 6H); 13C NMR (100 MHz, CDCl_3_) *δ*: 162.52, 146.83, 145.09, 143.42, 139.43, 132.30, 129.37 (q, *J*_C-F_ = 30.0 Hz), 129.22, 128.73 (q, *J*_C-F_ = 33.3 Hz), 128.65, 128.38, 125.94, 123.72 (q, *J*_C-F_ = 272.4 Hz, CF_3_), 123.50 (q, *J*_C-F_ = 270.4 Hz, CF_3_), 123.10, 123.06, 123.00, 106.52, 58.20, 57.03, 53.63, 43.85, 37.14, 35.08, 32.00, 28.36, 22.29; HR-MS *m/z* [M + H]^+^ (ESI^+^) calcd. for C_29_H_33_F_6_N_4_O = 567.2553, found 567.2549.

#### (R)-N-((1-(3-fluoro-4-(trifluoromethoxy)phenethyl)pyrrolidin-3-yl)methyl)-5-isobutyl-1-phenyl-1H-pyrazole-3-carboxamide (20l)

Yield 62%; ^1^H NMR (400 MHz, CDCl_3_) *δ*: 7.51–7.38 (m, 6H), 7.15 (td, *J* = 8.1, 0.9 Hz, 1H), 6.95 (dd, *J* = 11.0, 1.9 Hz, 1H), 6.90 (d, *J* = 8.4 Hz, 1H), 6.76 (s, 1H), 3.47–3.38 (m, 2H), 2.79–2.61 (m, 6H), 2.53–2.47 (m, 5H), 2.05–2.00 (m, 1H), 1.86–1.80 (m, 1H), 1.60–1.56 (m, 1H), 0.86 (d, *J* = 6.6 Hz, 6H); 13C NMR (100 MHz, CDCl_3_) *δ*: 162.54, 154.23 (d, *J*_C-F_ = 250.4 Hz), 146.80, 145.07, 141.37 (d, *J*_C-F_ = 6.4 Hz), 139.44, 134.58 (d, *J*_C-F_ = 1.9 Hz), 134.46 (d, *J*_C-F_ = 2.1 Hz), 129.22, 128.67, 125.92, 125.83, 124.66, 124.60, 124.53 (d, *J*_C-F_ = 6.9 Hz), 123.41, 123.32, 120.48 (q, *J*_C-F_ = 256.6 Hz, CF_3_), 117.25 (d, *J*_C-F_ = 18.1 Hz), 115.41 (d, *J*_C-F_ = 20.1 Hz), 112.89 (d, *J*_C-F_ = 19.4 Hz), 106.49, 58.14, 57.38, 57.08, 53.69, 43.74, 43.63, 37.10, 35.08, 34.91, 34.62, 28.28, 22.28.

#### (R)-5-isobutyl-1-phenyl-N-((1-(4-(trifluoromethoxy)-3-(trifluoromethyl)phenethyl)-pyrrolidin-3-yl)methyl)-1H-pyrazole-3-carboxamide (20m)

Yield 68%; ^1^H NMR (400 MHz, CDCl_3_) *δ*: 7.52 (t, *J* = 5.4 Hz, 1H), 7.48–7.38 (m, 6H), 7.34 (dd, *J* = 8.5, 1.8 Hz, 1H), 7.26 (d, *J* = 8.2 Hz, 1H), 6.76 (s, 1H), 3.49–3.37 (m, 2H), 2.82 (t, *J* = 7.9 Hz, 2H), 2.76–2.62 (m, 4H), 2.53–2.47 (m, 5H), 2.05–1.98 (m, 1H), 1.86–1.79 (m, 1H), 1.62–1.56 (m, 1H), 0.85 (d, *J* = 6.6 Hz, 6H); 13C NMR (100 MHz, CDCl_3_) *δ*: 162.53, 146.83, 145.03, 144.71, 139.46, 139.41, 133.46, 129.18, 128.63, 127.47 (q, *J*_C-F_ = 4.7 Hz), 125.87, 122.71 (q, *J*_C-F_ = 31.5 Hz), 122.49 (q, *J*_C-F_ = 271.3 Hz, OCF_3_), 120.89, 120.88, 120.26 (q, *J*_C-F_ = 257.5 Hz, CF_3_), 106.47, 58.28, 57.10, 53.71, 43.91, 36.99, 35.03, 34.62, 28.29, 28.26, 22.23; HR-MS *m/z* [M + H]^+^ (ESI^+^) calcd. for C_29_H_33_F_6_N_4_O_2_ = 583.2502, found 583.2497.

#### (R)-5-Isobutyl-N-((1-(4-methyl-3-(trifluoromethyl)phenethyl)pyrrolidin-3-yl)methyl)-1-phenyl-1H-pyrazole-3-carboxamide (20n)

Yield 89%; ^1^H NMR (400 MHz, CDCl_3_) *δ*: 7.49–7.37 (m, 7H), 7.20–7.14 (m, 2H), 6.77 (s, 1H), 3.48–3.40 (m, 2H), 2.81–2.61 (m, 6H), 2.56–2.47 (m, 5H), 2.42 (d, *J* = 1.6 Hz, 3H), 2.05–2.01 (m, 1H), 1.87–1.81 (m, 1H), 1.61–1.56 (m, 1H), 0.87 (d, *J* = 6.6 Hz, 6H); 13C NMR (100 MHz, CDCl_3_) *δ*: 162.50, 146.84, 144.97, 139.42, 138.00, 134.04, 131.84, 131.77, 129.28, 129.18, 128.63 (q, *J*_C-F_ = 29.3 Hz), 128.61, 125.87, 125.83, 125.77, 125.72, 124.59 (q, *J*_C-F_ = 272.0 Hz, CF_3_), 106.47, 58.33, 57.59, 53.76, 43.85, 37.03, 35.06, 34.81, 28.31, 22.26, 18.81; HR-MS *m/z* [M + H]^+^ (ESI^+^) calcd. for C_29_H_36_F_3_N_4_O = 513.2835, found 513.2826.

#### (R)-5-Isobutyl-N-((1-(2-methyl-5-(trifluoromethyl)phenethyl)pyrrolidin-3-yl)methyl)-1-phenyl-1H-pyrazole-3-carboxamide (20o)

Yield 69%; ^1^H NMR (400 MHz, CDCl_3_) *δ*: 7.49–7.39 (m, 6H), 7.34 (s, 2H), 7.20 (d, *J* = 8.4 Hz, 1H), 6.77 (s, 1H), 3.52–3.40 (m, 2H), 2.89–2.76 (m, 4H), 2.67–2.61 (m, 3H), 2.58–2.55 (m, 2H), 2.51 (d, *J* = 7.2 Hz, 2H), 2.32 (s, 3H), 2.11–2.03 (m, 1H), 1.89–1.78 (m, 1H), 1.67–1.59 (m, 1H), 0.86 (d, *J* = 6.6 Hz, 6H); 13C NMR (100 MHz, CDCl_3_) *δ*: 162.57, 146.76, 145.05, 140.30, 139.39, 138.92, 130.46, 129.20, 128.66, 128.25 (q, *J*_C-F_ = 31.6 Hz), 125.87, 125.75, 125.68, 125.64, 124.35 (q, *J*_C-F_ = 271.8 Hz, CF_3_), 122.96, 122.92, 106.49, 58.22, 56.17, 53.76, 43.64, 37.14, 35.06, 32.44, 28.27, 22.27, 19.30; HR-MS *m/z* [M + H]^+^ (ESI^+^) calcd. for C_29_H_36_F_3_N_4_O = 513.2835, found 513.2829.

#### (R)-5-Isobutyl-N-((1-(5-methyl-2-(trifluoromethyl)phenethyl)pyrrolidin-3-yl)methyl)-1-phenyl-1H-pyrazole-3-carboxamide (20p)

Yield 68%; ^1^H NMR (400 MHz, CDCl_3_) *δ*: 7.49–7.37 (m, 7H), 7.08–7.05 (m, 2H), 6.76 (s, 1H), 3.50–3.40 (m, 2H), 2.95–2.91 (m, 2H), 2.76–2.46 (m, 9H), 2.34 (s, 3H), 2.08–2.00 (m, 2H), 1.89–1.79 (m, 1H), 1.63–1.55 (m, 1H), 0.87 (d, *J* = 6.6 Hz, 6H); 13C NMR (100 MHz, CDCl_3_) *δ*: 162.50, 146.84, 144.98, 141.91, 139.42, 138.60, 132.24, 129.18, 128.59, 126.77, 125.87, 125.81 (q, *J*_C-F_ = 29.5 Hz), 125.80, 125.75, 124.77 (q, *J*_C-F_ = 271.7 Hz, CF_3_), 106.49, 58.24, 57.75, 53.62, 43.80, 37.12, 35.08, 32.00, 28.27, 22.28, 21.16.

### Biological assay methods

*FDSS6000 assay.* HEK 293 cells which express both stable Ca_V_3.1 and Ca_V_3.2 were grown in Dulbecco’s modified Eagle’s medium supplemented with 10% (v/v) fetal bovine serum, penicillin (100 μg/mL), streptomycin (100 μg/mL), geneticin (500 μg/mL), and puromycin (1 μg/mL) at 37 °C in a humid atmosphere of 5% CO_2_ and 95% air. Cells were seeded into 96-well black wall clear bottom plates at a density of 40,000 cells/well and were used on the next day for FDSS6000 assay. Cells were incubated for 60 min at room temperature with 5 μM fluo3/AM and 0.001% Pluronic F-127 in a HEPES-buffered solution composed of 115 mM NaCl, 5.4 mM KCl, 0.8 mM MgCl_2_, 1.8 mM CaCl_2_, 20 mM HEPES and 13.8 mM glucose (pH 7.4). During fluorescence-based FDSS6000 assay, Ca_V_3.1 and Ca_V_3.2 channels were activated using a high concentration of 70 mM KCl in 10 mM CaCl_2_ contained a HEPES-buffered solution, and the increase in [Ca^2+^]_i_ by KCl-induced depolarisation was detected. Throughout the entire procedure, cells were washed in a BIO-TEK 96-well washer. All data were collected and analysed using FDSS6000 and related software (Hamamatsu, Japan).

#### Whole-cell patch-clamp method

Ca_v_3.1: Ca_v_3.1 channel was stably expressed in HEK293 cells. The cells used for T-type calcium channel activity assay were cultured on a coverslip coated with poly-L-lysine (0.5 mg/mL) whenever sub-cultured, and their calcium channel activity was recorded 2–7 days after the cultivation. Current of the T-type calcium channel at a single cell level was measured according to an electrophysiological whole-cell patch-clamp method using EPC-9 amplifier (HEKA, Germany). At this time, a cell exterior solution composed of 140 mM NaCl, 2 mM CaCl_2_, and 10 mM HEPES (pH 7.4) and a cell interior solution composed of KCl 130 mM, HEPES 10 mM, EGTA 11 mM, and MgATP 5 mM (pH 7.4) were employed. Inward current caused by the T-type calcium channel activation which occurred when the cells were converted into a whole-cell recording mode by stabbing a microglass electrode having 3–4 MΩ resistance which was filled with the cell interior solution into a single cell and depolarised at –30 mV (50 ms duration period) every 10 s with fixing membrane potential to –100 mV was measured according to a T-type calcium channel protocol activated at low current. Ca_v_3.2: current of the calcium channel expressed from the *Xenopus* unfertilised oocytes was measured according to a two-electrode voltage clamp method. Current was measured in 10 mM Ba^2+^ solution composed of 10 mM Ba(OH)_2_, 90 mM NaOH, 1 mM KCl, 0.1 mM EDTA, and 5 mM HEPES, and pH was adjusted to 7.4 with methanesulfonic acid. At this time, voltage clamp and current measurements were regulated with an amplifier for unfertilised oocytes (Model OC-725C, Warner Instrument Corp., LLC., Hamden, CT, USA), analog signals were converted into digital signals using Digidata 2000 A (Analog-Digital converter, Axon Instrument), and acquisition, storage, and analysis of all data were recorded in Pentium IV computer via pCLAMP8. The data were mainly collected at 5 KHz and filtered at l KHz (Model 902 filter; Frequency devices located at Haverhill, MA, USA). The generation of T-type current was occurred by imposing test electric potential of –20 mV every 15 s on the unfertilised oocytes whose potential was fixed at –90 mV, and a blocking percentage was calculated by comparing the potentials before and after the drug treatment.

#### *In vivo* assay

Behavioural tests were conducted for rats at 1 day prior to surgery and 14 days after surgery. After the postoperative behavioural test, the animals were treated orally with 100 mg/kg compound **20n** or gabapentin. The tests were reevaluated at 1 h, 3 h, and 5 h after administration. Mechanical allodynia: Testing was performed according to methods described in the literature^45^. Rats were acclimated in a transparent plastic boxes permitting freedom of movement with a wire mesh floor to allow access to the planter surface of the hind paws. A von Frey filament (Stoelting, Wood Dale, IL) was applied 5 times (once every 3–4 s) to hind paw. Von Frey filaments were used to assess the 50% mechanical threshold for paw withdrawal. The 50% withdrawal threshold was determined by using the up-down method and formula given by Dixon^46^: 50% threshold =10^(^*^X^*^+^*^kd^*^)^/10^4^, where *X* is the value of the final von Frey hair used (in log units), *k* is the tabular value for the pattern of positive/negative responses modified from the same literature, and *d* is the mean difference between stimuli in log units (0.17). Cold allodynia: To quantify cold sensitivity of the paw, brisk paw withdrawal in response to acetone application was measured as reported previously^47^. The rat was placed under a transparent plastic box on a metal mesh floor and acetone was applied to the plantar surface of the hind paw. To do this, an acetone bubble was formed at the end of a small piece of polyethylene tubing which was connected to a syringe. The bubble was then gently touched to the heel. The acetone quickly spread over the proximal half of the plantar surface of the hind paw. The acetone was applied 5 times to the hind paw at 2 min interval. The frequency of paw withdrawal was expressed as a percentage [(no. of trials accompanied by brisk foot withdrawal/total no. of trials) × l00]. The results of behavioural tests are expressed as a %MPE. For example, paw withdrawal thresholds were converted to %MPE by the following formula, using a cutoff of 15 g (the threshold for normal rats) to define maximum possible effect: (post drug threshold-baseline threshold)/(cutoff-baseline threshold) × 100. %MPE values near 100 indicate normal mechanical thresholds (i.e. at or near 15 g), whereas values near 0 indicate allodynia. The result of cold allodynia was also expressed as %MPE.

## Results and discussion

### Inhibitor design

**Figure 1.**
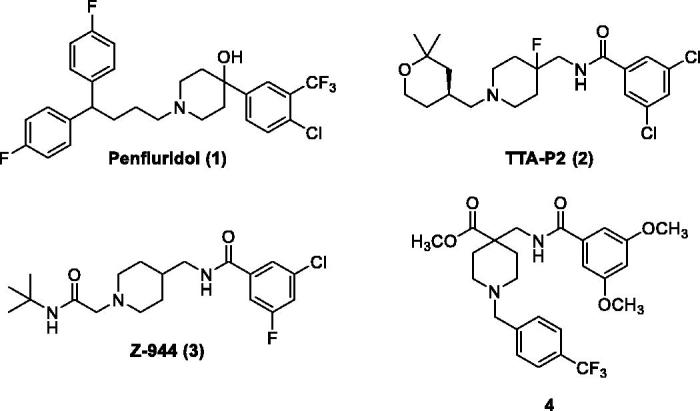
Previous T-type calcium channel inhibitors based on the piperidine scaffold.

**Figure 2.**
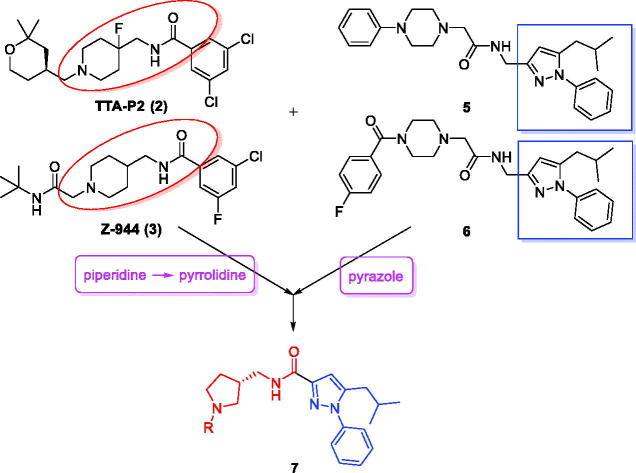
Design of new pyrrolidine-based T-type calcium channel inhibitors via a structure-hybridisation strategy.

**Figure 3.**
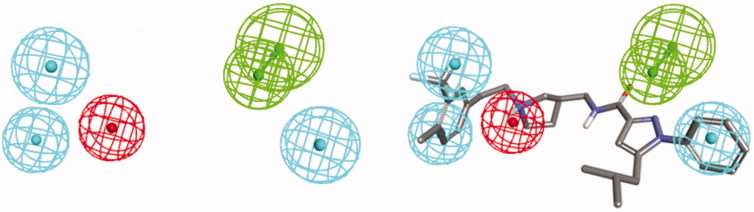
Left: Reference pharmacophore model. Right: pharmacophore mapping results of **20n**. Green: hydrogen bond acceptor, cyan: hydrophobic, red: positive ionisable.

Many promising T-type channel inhibitors have been developed based on a piperidine moiety such as Penfluridol (**1**)^48^, TTA-P2 (**2**) and Z-944 (**3**) (Figure 1). We also synthesised T-type channel inhibitors based on a piperidine moiety such as compound **4** which showed approximately 60% inhibition of Ca_v_3.1 at 10 μM^49^. However, pyrrolidine, a close homolog of piperidine, has not been frequently introduced so far. Therefore, in an effort to develop novel potent T-type channel inhibitors, we decided to synthesise pyrrolidine-based inhibitors. Our initial molecular structure was constructed via a structure-hybridisation between the template of TTA-P2 or Z-944 and 5-isobutyl-1-phenyl-1*H*-pyrazole moiety that consistently showed robust activity in our previous T-type inhibitors (Figure 2)^50^^,^^51^. Furthermore, this compound series accommodating hydrophobic R group are well mapped with 3D ligand-based pharmacophore that was produced by common feature hypothesis generation approach (HipHop) implemented in CATALYST program for the rational design of T-type calcium channel inhibitors (Figure 3)^52^.

### Synthesis of inhibitors

**Scheme 1.**
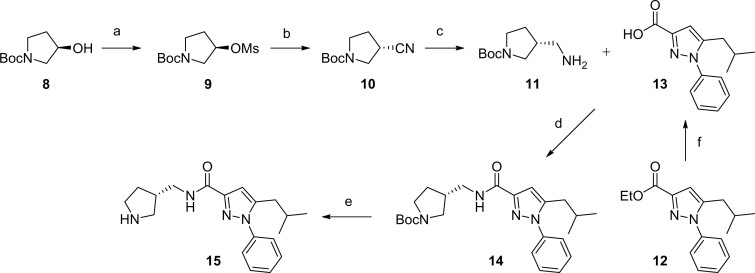
Reagents and conditions: (a) MsCl, TEA, DCM, rt, 100%; (b) NaCN, DMF, 80 °C, 84%; (c) 1.0 M LiAlH_4_ in THF, AlCl_3_, THF, 0 °C to rt, 56%; (d) 1,1d) % 56%lHaCN, DMFMFb) NaCN,rt, 90%; (e) TFA, DCM, rt, 94%; (f) 1 N NaOH, EtOH, rt, 82%.

We envisioned that the designed compounds would be obtained by an amide formation between (*N*-*tert*-butoxycarbonyl-pyrrolidin-3-yl)methanamines **11** and 5-isobutyl-1-phenyl-1*H*-pyrazole-3-carboxylic acid **13** followed by derivatisation of the free pyrrolidine **15**. The synthesis of the pyrroldine part commenced with mesylation of commercially available (*R*)-*N*-(*tert*-butoxycarbonyl)-3-hydroxypyrrolidine **8** (Scheme 1). Subsequent S_N_2 reaction with sodium cyanide followed by reduction with lithium aluminum hydride provided (*R*)-(*N*-(*tert*-butoxycarbonyl)-pyrrolidin-3-yl)methanamine **11** in good yield. 5-isobutyl-1-phenyl-1*H*-pyrazole-3-carboxylic acid **13** was produced by hydrolysis of the known ester **12**^53^ with 1 N NaOH. Next, carbonyldiimidazole-mediated amide formation smoothly provided the desired amide **14** in 90% yield, and the Boc group deprotection with TFA furnished the free amine **15** as a common synthetic intermediate.

**Scheme 2.**
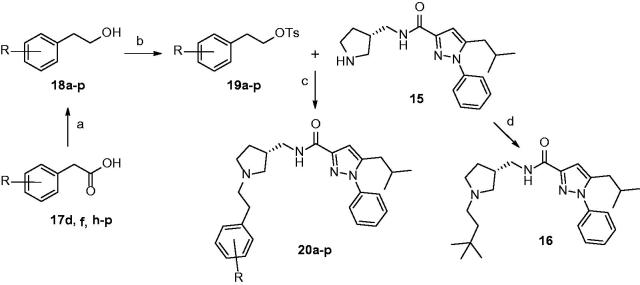
Reagents and conditions: (a) 1.0 M LiAlH_4_ in THF, THF, 0 °C to rt, 39–97%; (b) *p*-toluenesulfonyl chloride, TEA, DCM, 0 °C to rt, 12–87%; (c) K_2_CO_3_, acetonitrile, reflux, 17–89%; (d) 3,3-dimethylbutanal, NaBH(OAc)_3_, DCM, rt, 10–29%.

Preparation of *N*-alkylated pyrrolidines (**20a–p**) was started from synthesis of tosylates (**19a–p**): a variety of homobenzylic alcohol **18a–p** were either purchased or produced by reduction of commercially available homobenzylic acid **17d**, **17f**, and **17h–p** with lithium aluminum hydride (Scheme 2). After tosylation of **18a–p**, S_N_2 reaction with **15** underwent smoothly with potassium carbonate furnishing desired compounds **20a–p** in good yields. Additionally, *N*-(3,3-dimethybutyl)pyrrolidine **16** was obtained by reductive amination reaction with 3,3-dimethylbutanal.

### Biological activity

#### *In vitro* T-type calcium channel inhibitory activity

The *in vitro* T-type calcium channel inhibitory activity of synthesised pyrrolidine compounds were evaluated against Ca_V_3.1 and Ca_V_3.2 at two different concentrations, 10 μM and 1 μM, respectively (Table 1). We stably expressed Ca_V_3.1 and Ca_V_3.2 in HEK293 cells and measured the concentration of intracellular Ca^2+^ with fluo3/AM after the activation of Ca_V_3.1 and Ca_V_3.2 channels by 70 mM of KCl in 10 mM of CaCl_2_. Then, we presume that the degree of the fluorescence decrease read by FDSS6000 after co-incubation with synthesised compounds can reflect the inhibitory activity of each pyrrolidine compound^43^. First, the free pyrrolidine **15** was subjected to this FDSS6000-based fluorescent assay, but the compound was not able to inhibit both T-type channels more than 20% at 10 μM (18.60% inhibition against Ca_V_3.1 and 10.72% inhibition against Ca_V_3.2). Guided by the pharmacophore mapping results we decided to put a hydrophobic group to the free pyrrolidine to increase the potency. *N*-Alkylation of pyrrolidine dramatically increased inhibitory activities as exemplified with **16** and **20a** which inhibited the activation of Ca_V_3.1 and Ca_V_3.2 channels more than 47% at 10 μM. Since **20a** was more potent than **16**, we focused on the synthesis of *N*-phenethylpyrrolidine compounds. A variety of substituents such as methyl-, fluoro-, chloro-, trifluoromethyl- and trifluoromethoxy groups were introduced to the phenyl ring to investigate the substituent effects on the activity. Most compounds showed great inhibitory activities more than 65% against both channels at 10 μM, and 10 compounds (**20b–h**, **20 m**, **20n**, **20p**) inhibited greater than 80% of activation of at least one of either Ca_V_3.1 or Ca_V_3.2 channels. Furthermore, IC_50_ values of selected compounds were precisely measured by the whole-cell patch-clamp assay^44^, and compounds displaying high inhibition percentage at 10 μM also showed great IC_50_ values against both T-type calcium channels (IC_50_ (Ca_V_3.1) 2.14–6.11 μM, IC_50_ (Ca_V_3.2) 2.88–8.14 μM) strongly implicating that these pyrrolidine compounds possess great therapeutic potential for neuropathic pain.

**Table 1.** Inhibitory activities of pyrrolidine-based inhibitors **16** and **20a–p** against Ca_V_3.1 and Ca_V_3.2 T-type calcium channels.


Compounds	R	FDSS % inhibition				IC_50_ (μM)^a^	
		Ca_v_3.1 10 μM	Ca_v_3.1 1 μM	Ca_v_3.2 10 μM	Ca_v_3.2 1 μM	Ca_v_3.1	Ca_v_3.2
**16**	*t*-Bu	47.27	5.85	53.10	4.22	N.D.^b^	N.D.
**20a**	4-(trifluoromethyl)phenyl	65.50	13.08	66.06	1.07	N.D.	N.D.
**20b**	3-(trifluoromethyl)phenyl	76.65	19.12	85.34	21.53	N.D.	N.D.
**20c**	2-(trifluoromethyl)phenyl	71.14	22.43	81.64	29.40	N.D.	N.D.
**20d**	3-fluorophenyl	86.95	23.48	56.23	11.05	2.83 ± 0.42	3.59 ± 0.36
**20e**	3,4-difluorophenyl	89.51	21.39	71.63	14.47	3.20 ± 0.34	6.45 ± 0.52
**20f**	3,5-difluorophenyl	88.75	32.42	68.59	14.64	3.28 ± 0.31	5.99 ± 0.71
**20g**	2,3-difluorophenyl	87.42	18.81	65.18	2.66	3.42 ± 0.20	6.96 ± 0.93
**20h**	3-chloro-5-fluorophenyl	92.72	38.27	77.01	27.65	2.75 ± 0.20	5.07 ± 0.23
**20i**	4-chloro-3-fluorophenyl	68.88	10.20	68.83	14.13	6.11 ± 0.12	8.11 ± 0.70
**20j**	3,4-dichlorophenyl	71.16	19.93	69.29	19.82	5.95 ± 0.64	8.14 ± 0.75
**20k**	2,4-(ditrifluoromethyl)phenyl	75.56	23.93	69.08	8.89	N.D.	N.D.
**20l**	3-fluoro-4-(trifluoromethoxy)phenyl	67.30	11.57	69.41	15.98	N.D.	N.D.
**20m**	4-(trifluoromethoxy)-3-(trifluoromethyl)phenyl	93.22	36.29	72.11	25.48	2.14 ± 0.08	2.88 ± 0.23
**20n**	4-methyl-3-(trifluoromethyl)phenyl	81.42	21.81	74.53	16.84	3.36 ± 0.25	6.24 ± 0.65
**20o**	2-methyl-5-(trifluoromethyl)phenyl	74.84	17.34	71.67	15.86	N.D.	N.D.
**20p**	5-methyl-2-(trifluoromethyl)phenyl	81.51	25.66	79.18	23.09	N.D.	N.D.
**Mibefradil**	–	81.0	N.D.	76.0	N.D.	0.86 ± 0.14	0.28 ± 0.01

^a^Values represent the mean ± SD from triplicate experiments.

^b^Not determined.

#### *In vitro* ADME properties

In order to further evaluate the potential of these pyrrolidine compounds as promising therapeutic agents, their respective physicochemical properties were evaluated by assessing CYP450 inhibitory activities at 10 μM and metabolic stability in human liver microsome (Table 2). Most compounds barely inhibited CYP1A2 enzyme, but several compounds such as **20k** showed moderate to strong inhibition of other tested CYP enzymes. Also, we determined remaining compound percentage at 30 min after incubation of compounds in human liver microsome, but unfortunately the metabolic stability of synthesised pyrrolidines appeared not quite high. Nevertheless, among *N*-phenethylpyrrolidine compounds, **20n** seemed most stable in human liver microsome, approximately 15% of **20n** left intact after 30 min. As a next step, IC_50_ values of selected compounds against hERG channels were evaluated employing the same whole-cell patch-clamp assay (Table 2). Blockage of hERG channels induces long QT syndrome leading to potentially fatal cardiac arrhythmia^54^. Often, t-type calcium channel inhibitors suffer from low selectivity against hERG channels which we should investigate in developing t-type channel blockers to avoid potential cardiac side effects. The IC_50_ value of Mibefradil against hERG channels was reported as approximately 1.34 μM^49^. It was observed that most of our compounds showed lower IC_50_ values than Mibefradil requiring further improvement of selectivity against hERG channels. Fortunately, better selectivity against hERG channels than Mibefradil was obtained with compounds **16**, **20k** and **20n**, among which **20n** exhibited IC_50_ value of 8.13 μM representing safest compounds in this series of compounds. Comprehensive evaluation of current pyrrolidine compound series based on T-type channel inhibitory activity and *in vitro* ADME properties led us to determine **20n** as a promising candidate for the next *in vivo* evaluation.

**Table 2.** Results of *in vitro* ADME assay (CYP inhibition, microsomal stability, hERG inhibition) of pyrrolidine-based inhibitors **16** and **20a–p**.

Compounds	% Control of CYP-450 at 10 μM^a^					Stability in HLM^b,c^	hERG IC_50_ (μM)^d^
	2C19	1A2	2D6	2C9	3A4		
**16**	N.D.^e^	72.74	109.81	87.39	39.86	49.65	5.65 ± 0.64
**20a**	N.D.	53.24	29.42	16.85	6.46	14.04	0.32 ± 0.08
**20b**	52.81	79.27	26.69	41.5	40.04	10.16	N.D.
**20c**	75.7	81.01	35.83	35.83	39.83	3.65	N.D.
**20d**	51.67	100.26	66.21	71.12	43.91	6.01	N.D.
**20e**	26.67	98.62	55.37	71.07	32.67	5.04	N.D.
**20f**	42.78	118.04	50.95	69.93	41.12	1.14	N.D.
**20g**	37.22	87.76	70.63	73.99	28.70	3.08	N.D.
**20h**	39.44	109.61	45.58	49.27	38.24	7.14	0.93 ± 0.22
**20i**	1.35	68.90	34.89	27.85	68.23	1.61	0.84 ± 0.16
**20j**	1.85	62.81	26.03	21.7	57.48	5.20	0.75 ± 0.24
**20k**	22.39	95.54	5.20	12.41	19.98	12.41	5.23 ± 1.44
**20l**	33.59	91.99	34.05	70.19	59.05	2.88	0.31 ± 0.14
**20m**	36.11	80.68	60.42	10.15	42.99	3.52	N.D.
**20n**	22.27	121.46	23.21	17.34	17.32	14.65	8.13 ± 0.41
**20o**	43.11	125.86	17.52	27.67	18.29	13.17	1.70 ± 0.41
**20p**	43.98	63.63	28.57	55.04	23.72	4.29	N.D.

^a^Values represent the remaining % activities.

^b^Values represent % remaining after 30 min.

^c^HLM: human liver microsome.

^d^Values represent the mean ± SD from triplicate experiments.

^e^Not determined.

#### *In vivo* pharmacokinetic study

First, *in vivo* pharmacokinetic studies were carried out with Sprague-Dawley (SD) male rats (Table 3). Total eight rats were equally divided into two groups and 10 mg/kg of **20n** was administered to each group but in two different routes, orally and intravenously. Then, pharmacokinetic parameters of each administration route were calculated from compound concentrations in the blood. It turned out moderate *C*_max_ value (0.20 μg/mL) was observed after the oral administration. Nevertheless, because the clearance rate (29.7 ml/min/kg) was relatively low, total AUC_0-∞_ values after both oral and intravenous dosing were 339.17 and 61.89 μg min/mL sufficient enough to exert therapeutic effects *in vivo*. Of particular importance is the observation that brain-to-plasma ratio at 2 h was 8.78 after intravenous administration, strongly suggesting that once exposed to the plasma, **20n** is highly likely to cross the tight blood-brain barrier efficiently and well penetrated into the brain. Despite low oral bioavailability presumably caused by poor metabolic stability, **20n** can be employed to develop promising candidates for the treatment of neuropathic pain.

**Table 3.** Pharmacokinetic parameters of **20n** after intravenous and oral administration (10 mg/kg) to SD male rats.

Parameters	Intravenous	Oral
AUC_0–∞_ (μg min/mL)	339.17 ± 34.01	61.89 ± 27.81
AUC_last_ (μg min/mL)	273.03 ± 31.96	49.52 ± 20.31
Terminal half-life (min)	204.19 ± 26.24	193.03 ± 45.4
*C*_max_ (μg/mL)	–	0.20 ± 0.09
*T*_max_ (min)	–	68 (30 ∼ 120)^a^
CL (mL/min/kg)	29.71 ± 2.97	–
MRT (min)	130.34 ± 8.92	–
*V_ss_* (mL/kg)	7665.84 ± 1424.27	–
Plasma concentration (μg/mL) at 2 h	0.45 ± 0.03	0.16 ± 0.05
Brain concentration (μg/mL) at 2 h	3.9575 ± 0.2125	0.0211 ± 0.0062
Brain-to-plasma ratio (B/P) at 2 h	8.78	0.15
*F* (%)	18.2	

Values are presented as mean ± SD. AUC: area under the plasma concentration versus time curve); *C*_max_: peak plasma concentration; *T*_max_: time to reach *C*_max_; CL: time-averaged body clearance; MRT: mean residence time; *V*_ss_: apparent volume of distribution at steady state; *F*: oral bioavailability.

^a^Median (range) for *T*_max_.

#### *In vivo* efficacy studies with two neuropathic pain animal models

**Figure 4.**
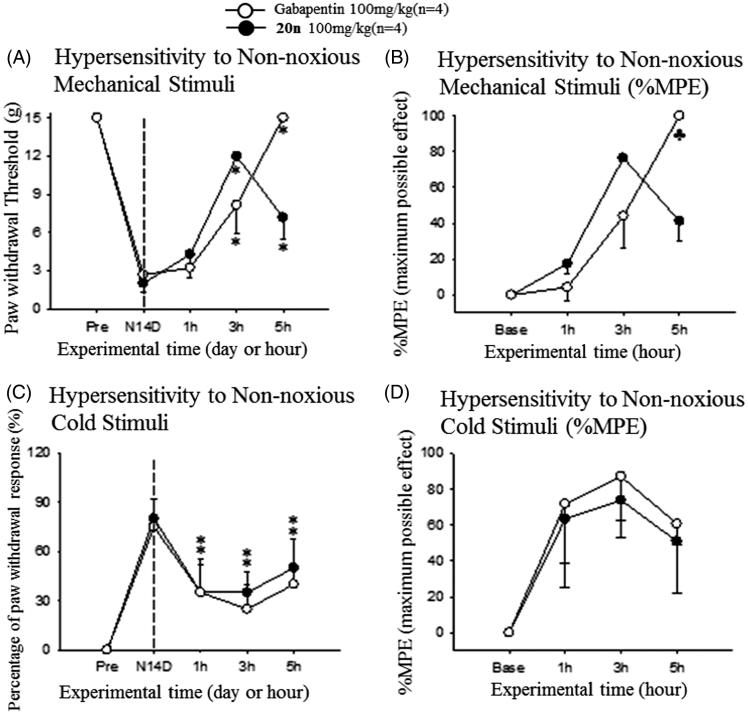
Analgesic effects to reduce hypersensitivity to non-noxious mechanical stimuli (A and B) and cold stimuli (C and D) after oral administration of gabapentin (○, 100 mg/kg, *n* = 4) and **20n** (•, 100 mg/kg, *n* = 4) to the SNL model. Experimental time expressed as D for days after neuropathic injury (N) and h for hours after gabapentin or **20n** administration, *p < .05 (gabapentin), *p < .05 (**20n**) versus pre-administration value (paired *t*-test), ♣*p* < .05 gabapentin versus **20n** (unpaired *t*-test).

In order to further demonstrate the potential of **20n** as a therapeutic agent to treat neuropathic pain, we tested *in vivo* efficacy of **20n** with two different animal models of neuropathic pain: the SNL model^55^ and the streptozotocin (STZ)-induced diabetic neuropathy model^56^. The SNL model is generated by ligating one (L5) or two (L5 and L6) segmental spinal nerves of the rat. In addition, streptozotocin is known to destroy pancreatic beta cells via DNA alkylation followed by activation of apoptosis resulting in diabetes. Both models result in behavioural symptoms such as increased sensitivity to non-noxious mechanical or cold stimuli and heat hypersensitivity, and have been widely used as animal model systems to investigate neuropathic pain. In the SNL model, we first observed that the paw withdrawal threshold to mechanical stimuli was significantly decreased and also the frequency of the paw withdrawal to cold stimuli was substantially increased confirming that neuropathic pain was induced 2 weeks after the surgery (Figure 4). Then, **20n** was orally administered to rats (*n* = 4) with 100 mg/kg. The current drug for neuropathic pain, gabapentin (100 mg/kg) was also dosed orally to another rat group (*n* = 4) side by side to directly compare pain-relieving effects with **20n**. The mechanical threshold was evaluated by von Frey filaments. It was found that **20n** was able to restore mechanical hypersensitivity close to the normal level and showed superior analgesic effects as compared to gabapentin at 3 h after the treatment. Furthermore, **20n** significantly lowered the paw withdrawal response rate to cold stimuli as well, and the analgesic effects of **20n** were again similar to that of gabapentin.

**Figure 5.**
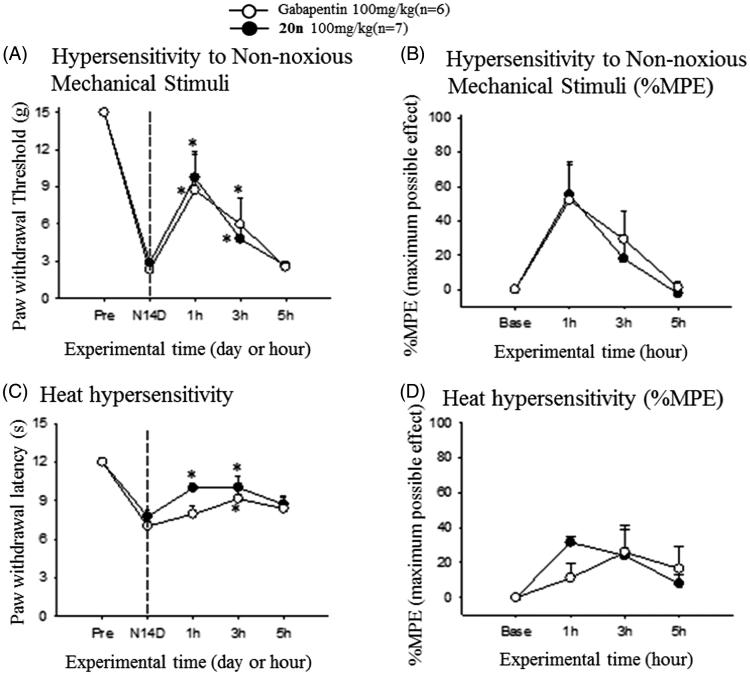
Analgesic effects to reduce hypersensitivity to non-noxious mechanical stimuli (A and B) and heat hypersensitivity (C and D) after oral administration of gabapentin (○, 100 mg/kg, *n* = 6) and **20n** (•, 100 mg/kg, *n* = 7) to the STZ model. Experimental time expressed as D for days after neuropathic injury (N) and h for hours after gabapentin or **20n** administration, *p < .05 (gabapentin), *p < .05 (**20n**) versus pre-administration value (paired *t*-test), ♣*p* < .05 gabapentin versus **20n** unpaired *t*-test).

After observing treatment of **20n** relieved neuropathic pain response in the SNL model, we decided to further prove that the ability of **20n** to reduce neuropathy can be general and are not confined to one specific neuropathic model. The streptozotocin (STZ)-induced diabetic neuropathy model for diabetic peripheral neuropathy (DPN) was selected. We confirmed that intraperitoneal injection of 65 mg/kg of streptozotocin to rats reduced both the paw withdrawal threshold to non-noxious mechanical stimuli and the paw withdrawal latency to heat stimuli at 2 weeks after the surgery (Figure 5). Subsequently, 100 mg/kg of **20n** and gabapentin was orally dosed to seven and six rats, respectively. Once again, **20n** increased the paw withdrawal threshold similarly to gabapentin, and delayed the paw withdrawal latency comparable to gabapentin suggesting **20n** is also effective in alleviating heat hypersensitivity.

## Conclusion

In conclusion, we have developed pyrrolidine-based T-type calcium channel inhibitors by pharmacophore mapping and structural hybridisation followed by evaluation of their Ca_v_3.1 and Ca_v_3.2 channel inhibitory activities with FDSS and whole-cell patch-clamp assay. Most pyrrolidine compounds potently inhibited activation of both Ca_v_3.1 and Ca_v_3.2 T-type channels showing IC_50_ values between 2.14 and 8.14 μM. Profiling of *in vitro* ADME properties revealed that among this series **20n** has great selectivity against hERG channel and high plasma exposure in the subsequent *in vivo* pharmacokinetic study. Furthermore, it was notable that **20n** can be potentially well penetrated into the brain according to the excellent brain to plasma compound concentration ratio at 2 h after intravenous administration. Finally, **20n** exhibited comparable analgesic effects to gabapentin in the both SNL and STZ neuropathic pain animal models, implicating potent T-type calcium channel inhibitor **20n** can be developed as promising candidates to treat neuropathic pain. Further optimisation of **20n** in regard to physicochemical properties like metabolic stability is currently in progress.

## Supplementary Material

Supplemental Material
