# Supplementary material for: Synthesis and biological evaluation of pyrrolidine-based T-type calcium channel inhibitors for the treatment of neuropathic pain
Source: J Enzyme Inhib Med Chem. 2018 Sep 20;33(1):1460–71. doi: 10.1080/14756366.2018.1513926 (PMC6151954; doi:10.1080/14756366.2018.1513926)
Supplement: Supplemental Material [file IENZ_A_1513926_SM9124.pdf]

Synthesis and Biological Evaluation of Pyrrolidine-based T-type Calcium Channel Inhibitors for the Treatment of Neuropathic Pain

Hak Kyun Yang[a,1], Woo Seung Son[a,c,1], Keon Seung Lim[d], Gun Hee Kim[e], Eun Jeong Lim[a], Changdev G. Gadhe[a], Jae Yeol Lee[e], Kyu-Sung Jeong[c], Sang Min Lim[a,f]*, Ae Nim Pae[a,b]*

[a]*Convergence Research Center for Diagnosis, Treatment and Care System of Dementia, Korea Institute of Science and Technology, Seoul 02792, Republic of Korea*

[b]*Division of Bio-Medical Science & Technology, KIST School, Korea University of Science and Technology, Seoul 02792, Republic of Korea*

[c]*Department of Chemistry, Yonsei University, Seoul 03722, Republic of Korea*

[d]*1ST Biotherapeutics Inc., Seongnam, Gyeonggi-do 13493, Republic of Korea*

[e]*Research Institute for Basic Sciences and Department of Chemistry, College of Sciences, Kyung Hee University, Seoul 02447, Republic of Korea*

[f]*Division of Bio-Medical Science and Technology, Korea University of Science and Technology, Daejon 34113, Republic of Korea*

1. Synthesis of Intermediates

1.1. (*R*)-*tert*-butyl 3-(methylsulfonyloxy)pyrrolidine-1-carboxylate (**9**).

MsCl (1.5 ml, 19.38 mmol) and TEA (2.9 ml, 20.79 mmol) were slowly added dropwise to a solution of (*R*)-(-)-*N*-Boc-3-pyrrolidinol **8** (3.0 g, 16.02 mmol) in anhydrous DCM at 0 °C. The mixture was stirred at room temperature for 7 h. It was quenched with water and brine, extracted with DCM. It was dried over magnesium sulfate and concentrated in vacuo. The residue was purified via column chromatography on silica gel (*n*-hexane/ethyl acetate = 1:1), the desired compound (4.25 g) was obtained. (yield: 100%); ^{1}H NMR (300 MHz, $CDCl_3$) δ 5.24-5.20 (m, 1H), 3.63-3.41 (m, 4H), 3.01 (s, 3H), 2.22-2.10 (m, 2H), 1.42 (s, 9H).

1.2. (*S*)-*tert*-butyl-3-cyanopyrrolidine-1-carboxylate (**10**)

NaCN (3.14 g, 64.07 mmol) was added to a solution of (*R*)-*tert*-butyl 3-(methylsulfonyloxy) pyrrolidine-1- carboxylate **9** (4.25 g, 16.02 mmol) in DMF. The mixture was stirred at 80 °C for 12 h. It was quenched with water and extracted with DCM. It was dried over magnesium sulfate and concentrated in vacuo. The residue was purified via column chromatography on silica gel (*n*-hexane/ethyl acetate= 3:1), the desired compound (2.61 g) was obtained. (yield: 84%); ^{1}H NMR (300 MHz, $CDCl_3$) δ 3.67-3.44 (m, 4H), 3.12-3.05 (m, 1H), 2.31-2.22 (m, 2H), 1.48 (s, 9H).

1.3. (*R*)-*tert*-butyl-3-(aminomethyl)pyrrolidine-1-carboxylate (**11**)

A solution of $AlCl_3$ (1.25 g, 9.37 mmol) in THF was added dropwise to a solution of 1.0 M $LiAlH_4$ (11.20 mL, 11.20 mmol) in THF. The mixture was stirred at room temperature for 20 min. After 20 min, a solution of (*S*)-*tert*-butyl-3-cyanopyrrolidine-1-carboxylate **10** (2.00 g, 9.98 mmol) in THF was slowly added dropwise to the mixture at 0 °C. The reaction mixture was stirred at room temperature for 1 h. It was quenched with MeOH, concentrated in vacuo.

The residue was acidified with 1 N HCl and extracted with EA. The aqueous layer was basic with 10 N NaOH and extracted with DCM. It was dried over sodium sulfate and concentrated in vacuo. The desired compound (1.25 g) was obtained. (yield: 61%); ^{1}H NMR (300 MHz, $CDCl_3$) δ 3.55-3.30 (m, 4H), 3.02-3.00 (m, 1H), 2.73 (t, J = 6.2 Hz, 2H), 2.24 (br s, 1H), 2.01-1.98 (m, 1H), 1.60-1.57 (m, 1H), 1.26 (s, 1H).

1.4. 5-Isobutyl-1-phenyl-1*H*-pyrazole-3-carboxylic acid (**13**)

1 N aqueous NaOH (25.61 mL, 25.61 mmol) was added dropwise to a solution of ethyl 5-isobutyl-1-phenyl-1*H*-pyrazole-3-carboxylate **12** (2.79 g, 10.24 mmol) in ethanol (30 mL). The mixture was stirred at room temperature for 1 h. The reaction mixture was evaporated and quenched with water. The aqueous layer was acidified with 1 N aqueous HCl, filtered and dried in vacuo. The desired compound (2.05 g) was obtained. (yield: 82%); ^{1}H NMR (300 MHz, $CDCl_3$) δ 7.42–7.30 (m, 5H), 6.81 (s, 1H), 2.52 (d, J = 7.2 Hz, 2H), 1.92–1.79 (m, 1H), 0.89 (d, J = 6.6 Hz, 6H).

1.5. (*R*)-*tert*-Butyl 3-((5-isobutyl-1-phenyl-1*H*-pyrazole-3-carboxamido)methyl) pyrrolidine-1-carboxylate (**14)**

1,1'-Carbonyldiimidazole (1.45 g, 8.93 mmol) in anhydrous THF was added dropwise to a solution of 5-Isobutyl-1-phenyl-1*H*-pyrazole-3-carboxylic acid **13** (2.00 g, 8.19 mmol) in anhydrous THF (20 mL). The mixture was stirred at room temperature for 2 h. After 2 h, (*R*)-*tert*-Butyl 3-(aminomethyl)pyrrolidine-1-carboxylate (1.49 g, 7.44 mmol) **11** in anhydrous THF was added dropwise to the mixture and stirred at room temperature for 3 h. The reaction mixture was evaporated and quenched with an aqueous solution of saturated sodium bicarbonate and water. The aqueous layer was extracted with ethyl acetate. The combined organic layer was dried over sodium sulfate and concentrated in vacuo. The residue was purified via column chromatography on silica gel (ethyl acetate/hexane = 1:2, R_f= 0.3), the

desired compound (3.05 g) was obtained. (yield: 96%); ^{1}H NMR (300 MHz, $CDCl_3$) δ 7.56–7.41 (m, 5H), 7.08 (t, J = 6.0 Hz, 1H), 6.77 (s, 1H), 3.56–3.32 (m, 5H), 3.14–3.06 (m, 1H), 2.53 (d, J = 7.2 Hz, 3H), 2.07–2.00 (m, 1H), 1.91–1.84 (m, 1H), 1.71–1.68 (m, 1H), 1.47 (s, 9H), 0.89 (d, J = 6.6 Hz, 6H).

1.6. General procedure for the preparation of compounds **18d, f, h-p**

1.0 M solution of LAH in THF was added dropwise to a solution of the carboxylic acid in anhydrous THF in an ice bath. The reaction mixture was stirred at room temperature for 2 h, and then cooled to 0°C. The reaction was quenched with water and extracted with ethyl acetate. The organic layer was washed with brine, dried over sodium sulfate and concentrated in vacuo. The desired compound was obtained.

1.6.1. 2-(3-Fluorophenyl)ethanol (**18d**)

Following the general procedure, 2-(3-fluorophenyl)acetic acid **17d** (500 mg, 3.24 mmol) in anhydrous THF (10 mL) and 1.0 M solution of LAH in THF (4.87 mL, 4.87 mmol) gave the desired compound **18d** (425 mg, 94%); ^{1}H NMR (400 MHz, $CDCl_3$) δ 7.31–7.29 (m, 1H), 7.04 (d, J = 7.6 Hz, 1H), 6.99–6.96 (m, 1H), 3.90 (t, J = 6.4 Hz, 2H), 2.90 (t, J = 6.4 Hz, 2H), 1.48 (br, 1H).

1.6.2. 2-(3,5-Difluorophenyl)ethanol (**18f**)

Following the general procedure, 2-(3,5-difluorophenyl)acetic acid **17f** (300 mg, 1.74 mmol) in anhydrous THF (6 mL) and 1.0 M solution of LAH in THF (2.61 mL, 2.61 mmol) gave the desired compound **18f** (252 mg, 92%); ^{1}H NMR (400 MHz, $CDCl_3$) δ 6.79 (dd, J = 8.0, 2.0 Hz, 2H), 6.73–6.68 (m, 1H), 3.90 (t, J = 6.4 Hz, 2H), 2.88 (t, J = 6.4 Hz, 2H).

1.6.3. 2-(3-Chloro-5-fluorophenyl)ethanol (**18h**)

Following the general procedure, 2-(3-chloro-5-fluorophenyl)acetic acid **17h** (300 mg, 1.59 mmol) in anhydrous THF (7 mL) and 1.0 M solution of LAH in THF (2.39 mL, 2.39 mmol) gave the desired compound **18h** (264 mg, 95%); ^{1}H NMR (400 MHz, $CDCl_3$) δ 7.06 (d, J = 1.2 Hz, 1H), 6.99 (dt, J = 8.4, 2.0 Hz, 1H), 6.90–6.87 (m, 1H), 3.88 (t, J = 6.0 Hz, 2H), 2.86 (t, J = 6.4 Hz, 2H).

1.6.4. 2-(4-Chloro-3-fluorophenyl)ethanol (**18i**)

Following the general procedure, 2-(4-chloro-3-fluorophenyl)acetic acid **17i** (500 mg, 2.65 mmol) in anhydrous THF (10 mL) and 1.0 M solution of LAH in THF (3.98 mL, 3.98 mmol) gave the desired compound **18i** (427 mg, 92%); ^{1}H NMR (400 MHz, $CDCl_3$) δ 7.35 (t, J = 8.0 Hz, 1H), 7.07 (dd, J = 10.0, 2.0 Hz, 1H), 6.99 (dd, J = 8.0, 1.2 Hz, 1H), 3.89 (t, J = 6.4 Hz, 2H), 2.87 (t, J = 6.4 Hz, 2H), 1.49 (s, 1H).

1.6.5. 2-(3,4-Dichlorophenyl)ethanol (**18j**)

Following the general procedure, 2-(3,4-dichlorophenyl)acetic acid **17j** (250 mg, 1.22 mmol) in anhydrous THF (5 mL) and 1.0 M solution of LAH in THF (1.83 mL, 1.83 mmol) gave the desired compound **18j** (207 mg, 89%); ^{1}H NMR (400 MHz, $CDCl_3$) δ 7.41 (d, J = 8.2 Hz, 1H), 7.37 (d, J = 2.0 Hz, 1H), 7.10 (dd, J = 8.2, 2.1 Hz, 1H), 3.89 (t, J = 6.4 Hz, 2H), 2.85 (t, J = 6.4 Hz, 2H).

1.6.6. 2-(2,4-Bis(trifluoromethyl)phenyl)ethanol (**18k**)

Following the general procedure, 2-(2,4-bis(trifluoromethyl)phenyl)acetic acid **17k** (250 mg, 0.92 mmol) in anhydrous THF (5 mL) and 1.0 M solution of LAH in THF (1.38 mL, 1.38 mmol) gave the desired compound **18k** (93 mg, 39%); ^{1}H NMR (400 MHz, $CDCl_3$) δ 7.92 (s, 1H), 7.76 (d, J = 8.0 Hz, 1H), 7.59 (d, J = 8.4 Hz, 1H), 3.91 (t, J = 6.8 Hz, 2H), 3.13 (t, J = 6.8 Hz, 2H), 1.96 (s, 1H).

1.6.7. 2-(3-Fluoro-4-(trifluoromethoxy)phenyl)ethanol (**18l**)

Following the general procedure, 2-(3-fluoro-4-(trifluoromethoxy)phenyl)acetic acid **17l** (350 mg, 1.47 mmol) in anhydrous THF (8 mL) and 1.0 M solution of LAH in THF (2.20 mL, 2.20 mmol) gave the desired compound **18l** (320 mg, 97%); ^{1}H NMR (400 MHz, $CDCl_3$) *δ* 7.29–7.24 (m, 1H), 7.13 (dd, *J* = 10.8, 1.6 Hz, 1H), 7.05 (dd, *J* = 8.4, 1.2 Hz, 1H), 3.90 (t, *J* = 6.4 Hz, 2H), 2.89 (t, *J* = 6.4 Hz, 2H).

1.6.8. 2-(4-(Trifluoromethoxy)-3-(trifluoromethyl)phenyl)ethanol (**18m**)

Following the general procedure, 2-(4-(trifluoromethoxy)-3-(trifluoromethyl)phenyl)acetic acid **17m** (200 mg, 0.69 mmol) in anhydrous THF (5 mL) and 1.0 M solution of LAH in THF (1.04 mL, 1.04 mmol) gave the desired compound **18m** (175 mg, 93%); ^{1}H NMR (400 MHz, $CDCl_3$) *δ*7.57 (d, *J* = 1.9 Hz, 1H), 7.47 (dd, *J* = 5.4 Hz, 1H), 7.35 (d, *J* = 8.4 Hz, 1H), 3.91 (t, *J* = 6.4 Hz, 2H), 2.92 (t, *J* = 6.4 Hz, 2H).

1.6.9. 2-(4-Methyl-3-(trifluoromethyl)phenyl)ethanol (**18n**)

Following the general procedure, 2-(4-methyl-3-(trifluoromethyl)phenyl)acetic acid **17n** (500 mg, 2.29 mmol) in anhydrous THF (10 mL) and 1.0 M solution of LAH in THF (3.44 mL, 3.44 mmol) gave the desired compound **18n** (448 mg, 96%); ^{1}H NMR (300 MHz, $CDCl_3$) *δ* 7.50 (s, 1H), 7.34–7.23 (m, 2H), 3.01 (t, *J* = 6.3 Hz, 2H), 2.91 (t, *J* = 6.6 Hz, 2H), 2.49 (s, 3H), 1.49 (s, 1H).

1.6.10. 2-(2-Methyl-5-(trifluoromethyl)phenyl)ethanol (**18o**)

Following the general procedure, 2-(2-methyl-5-(trifluoromethyl)phenyl)acetic acid **17o** (200 mg, 0.92 mmol) in anhydrous THF (5 mL) and 1.0 M solution of LAH in THF (1.38 mL, 1.38 mmol) gave the desired compound **18o** (174 mg, 93%); ^{1}H NMR (400 MHz, $CDCl_3$) *δ* 7.45 (s, 1H), 7.41 (d, *J* = 8.0 Hz, 1H), 7.29 (d, *J* = 6.8 Hz, 1H), 3.90 (t, *J* = 6.8 Hz, 2H), 2.96

(t, J = 6.8 Hz, 2H), 2.41 (s, 3H), 1.45 (s, 1H).

1.6.11. 2-(5-Methyl-2-(trifluoromethyl)phenyl)ethanol (**18p**)

Following the general procedure, 2-(5-methyl-2-(trifluoromethyl)phenyl)acetic acid **17p** (450 mg, 2.06 mmol) in anhydrous THF (8 mL) and 1.0 M solution of LAH in THF (3.09 mL, 3.09 mmol) gave the desired compound **18p** (277 mg, 66%); ^{1}H NMR (400 MHz, $CDCl_3$) δ 7.55 (d, J = 8.0 Hz, 1H), 7.23 (s, 1H), 7.15 (d, J = 8.0 Hz, 1H), 3.90 (t, J = 6.8 Hz, 2H), 3.06 (t, J = 6.8 Hz, 2H), 2.41 (s, 3H).

1.7. General procedure for the preparation of compounds **19a-p**

Triethylamine (TEA) was added dropwise to a solution of the alcohol in anhydrous dichloromethane (DCM). The reaction mixture was stirred at room temperature for 1 h, and then cooled to 0 °C. 4-Toluenesulfonyl chloride in anhydrous DCM was added dropwise to the reaction mixture in an ice bath. The reaction mixture was stirred at room temperature for 5 h, and then quenched with water and extracted with DCM. The organic layer was washed with an aqueous solution of saturated sodium bicarbonate and brine. It was dried over sodium sulfate and concentrated in vacuo. The residue was purified via column chromatography on silica gel, the desired compound was obtained.

1.7.1. 4-(trifluoromethyl)phenethyl-4-methylbenzenesulfonate (**19a**)

Following the general procedure, 4-(trifluoromethyl)phenyl)ethanol **18a** (0.48 mL, 3.16mmol), TEA (0.89 mL, 6.32 mmol) and TsCl (2.1 g, 11.06 mmol) gave the desired compound **19a** (847 mg, 78%); ^{1}H NMR (300 MHz, $CDCl_3$) δ 7.64 (d, J = 8.3 Hz, 2H), 7.49 (d, J = 8.0 Hz, 2H), 7.25-7.21 (m, 4H), 4.26 (t, J = 6.6 Hz, 2H), 3.02 (t, J = 6.5 Hz, 2H), 2.44 (s, 3H).

1.7.2. 3-(trifluoromethyl)phenethyl-4-methylbenzenesulfonate (**19b**)

Following the general procedure, 3-(trifluoromethyl)phenyl)ethanol **18b** (462 mg, 2.43 mmol), TEA (0.68 mL, 4.86 mmol) and TsCl (1.62 g, 8.50 mmol) gave the desired compound **19b** (487 mg, 58%); ^{1}H NMR (300 MHz, $CDCl_3$) δ 7.65 (d, J = 4.2 Hz, 2H), 7.47-7.38 (m, 2H), 7.33-7.31 (m, 2H), 7.27-7.26 (m, 2H), 4.24 (t, J = 3.3 Hz, 2H), 3.01 (t, J = 3.3 Hz, 2H), 2.42 (s, 3H).

1.7.3. 2-(trifluoromethyl)phenethyl-4-methylbenzenesulfonate (**19c**)

Following the general procedure, 2-(trifluoromethyl)phenyl)ethanol **18c** (410 mg, 2.16 mmol), TEA (0.61 mL, 4.31 mmol) and TsCl (1.44 g, 7.54 mmol) gave the desired compound **19c** (89 mg, 12%); ^{1}H NMR (300 MHz, $CDCl_3$) δ 7.70 (d, J = 4.0 Hz, 2H), 7.59 (m, 1H), 7.46-7.45 (m, 1H), 7.35-7.32 (m, 2H), 7.30-7.26 (m, 2H), 4.22 (t, J = 3.3 Hz, 2H), 3.14 (t, J = 3.3 Hz, 2H), 2.43 (s, 3H).

1.7.4. 3-Fluorophenethyl 4-methylbenzenesulfonate (**19d**)

Following the general procedure, 2-(3-fluorophenyl)ethanol **18d** (411 mg, 2.93mmol), TEA (2.03 mL, 14.65 mmol) and TsCl (1.40 g, 7.33 mmol) gave the desired compound **19d** (714 mg, 83%); ^{1}H NMR (400 MHz, $CDCl_3$) δ 7.71 (d, J = 8.3 Hz, 2H), 7.32 (d, J = 8.0 Hz, 2H), 7.27–7.21 (m, 1H), 6.96–6.91 (m, 2H), 6.82-6.78 (m, 1H), 4.24 (t, J = 6.8 Hz, 2H), 2.97 (t, J = 6.8 Hz, 2H), 2.46 (s, 3H).

1.7.5. 3,4-Difluorophenethyl 4-methylbenzenesulfonate (**19e**)

Following the general procedure, 2-(3,4-difluorophenyl)ethanol **18e** (250 mg, 1.58 mmol), TEA (1.09 mL, 7.90 mmol) and TsCl (753 mg, 3.95 mmol) gave the desired compound **19e** (429 mg, 87%); ^{1}H NMR (400 MHz, $CDCl_3$) δ 7.70 (d, J = 8.3 Hz, 2H), 7.32 (d, J = 8.0 Hz, 2H), 7.08–7.02 (m, 1H), 6.91–6.84 (m, 2H), 4.22 (t, J = 6.4 Hz, 2H), 2.93 (t, J = 6.8 Hz, 2H), 2.47 (s, 3H).

1.7.6. 3,5-Difluorophenethyl 4-methylbenzenesulfonate (**19f**)

Following the general procedure, 2-(3,5-difluorophenyl)ethanol **18f** (242 mg, 1.53 mmol), TEA (1.06 mL, 7.65 mmol) and TsCl (730 mg, 3.83 mmol) gave the desired compound **19f** (359 mg, 75%); ^{1}H NMR (400 MHz, $CDCl_3$) δ 7.71 (d, J = 8.3 Hz, 2H), 7.33 (d, J = 8.0 Hz, 2H), 6.71–6.61 (m, 3H), 4.24 (t, J = 6.4 Hz, 2H), 2.95 (t, J = 6.4 Hz, 2H), 2.47 (s, 3H).

1.7.7. 2,3-Difluorophenethyl 4-methylbenzenesulfonate (**19g**)

Following the general procedure, 2-(2,3-difluorophenyl)ethanol **18g** (250 mg, 1.58 mmol), TEA (1.09mL, 7.90mmol) and TsCl (753mg, 3.95mmol) gave the desired compound **19g** (423 mg, 86%); ^{1}H NMR (400 MHz, $CDCl_3$) δ 7.70 (d, J = 8.4 Hz, 2H), 7.31 (d, J = 8.0 Hz, 2H), 7.09–6.92 (m, 3H), 4.26 (t, J = 6.8 Hz, 2H), 3.04 (t, J = 6.6 Hz, 2H), 2.46 (s, 3H).

1.7.8. 3-Chloro-5-fluorophenethyl 4-methylbenzenesulfonate (**19h**)

Following the general procedure, 2-(3-chloro-5-fluorophenyl)ethanol **18h** (257 mg, 1.47 mmol), TEA (1.02 mL, 7.35 mmol) and TsCl (702 mg, 3.68 mmol) gave the desired compound **19h** (356 mg, 74%); ^{1}H NMR (400 MHz, $CDCl_3$) δ 7.70 (d, J = 8.0 Hz, 2H), 7.32 (d, J = 8.0 Hz, 2H), 6.96 (dt, J = 8.4, 2.1 Hz, 1H), 6.88 (s, 1H), 6.73 (dt, J = 9.0, 1.8 Hz, 1H), 4.24 (t, J = 6.5 Hz, 2H), 2.94 (t, J = 6.5 Hz, 2H), 2.47 (s, 3H).

1.7.9. 4-Chloro-3-fluorophenethyl 4-methylbenzenesulfonate (**19i**)

Following the general procedure, 2-(4-chloro-3-fluorophenyl)ethanol **18i** (415 mg, 2.38 mmol), TEA (1.99 mL, 14.28 mmol) and TsCl (1.36 g, 7.13 mmol) gave the desired compound **19i** (653 mg, 83%); ^{1}H NMR (400 MHz, $CDCl_3$) δ 7.67 (d, J = 8.3 Hz, 2H), 7.31–7.25 (m, 3H), 6.87–6.83 (m, 2H), 4.23 (t, J = 6.4 Hz, 2H), 2.93 (t, J = 6.4 Hz, 2H), 2.47 (s, 3H).

1.7.10. 3,4-Dichlorophenethyl 4-methylbenzenesulfonate (**19j**)

Following the general procedure, 2-(3,4-dichlorophenyl)ethanol **18j** (202 mg, 1.06 mmol), TEA (0.89 mL, 6.36 mmol) and TsCl (604 mg, 3.17 mmol) gave the desired compound **19j** (320 mg, 87%); ^{1}H NMR (400 MHz, $CDCl_3$) δ 7.66 (d, J = 8.3 Hz, 2H), 7.33–7.28 (m, 3H), 7.14 (d, J = 2.0 Hz, 1H), 6.97 (dd, J = 8.2, 2.1 Hz, 1H), 4.23 (t, J = 6.4 Hz, 2H), 2.91 (t, J = 6.4 Hz, 2H), 2.47 (s, 3H).

1.7.11. 2,4-Bis(trifluoromethyl)phenethyl 4-methylbenzenesulfonate (**19k**)

Following the general procedure, 2-(2,4-bis(trifluoromethyl)phenyl)ethanol **18k** (114 mg, 0.44 mmol), TEA (0.37 mL, 2.64 mmol) and TsCl (252 mg, 1.32 mmol) gave the desired compound **19k** (120 mg, 66%); ^{1}H NMR (400 MHz, $CDCl_3$) δ 7.86 (s, 1H), 7.74 (m, 3H), 7.51 (d, J = 8.1 Hz, 1H), 7.31–7.31 (m, 2H), 4.28 (t, J = 6.4 Hz, 2H), 3.24 (t, J = 6.5 Hz, 2H), 2.45 (s, 3H).

1.7.12. 3-Fluoro-4-(trifluoromethoxy)phenethyl 4-methylbenzenesulfonate (**19l**)

Following the general procedure, 2-(3-fluoro-4-(trifluoromethoxy)phenyl)ethanol **18l** (299 mg, 1.33 mmol), TEA (0.92 mL, 6.65 mmol) and TsCl (635 mg, 3.33 mmol) gave the desired compound **19l** (344 mg, 68%); ^{1}H NMR (400 MHz, $CDCl_3$) δ 7.70 (d, J = 8.3 Hz, 2H), 7.32 (d, J = 8.0 Hz, 2H), 7.20 (t, J = 7.7 Hz, 1H), 6.94 (d, J = 8.5 Hz, 2H), 4.25 (t, J = 6.5 Hz, 2H), 2.98 (t, J = 6.5 Hz, 2H), 2.47 (s, 3H).

1.7.13. 4-(Trifluoromethoxy)-3-(trifluoromethyl)phenethyl 4-methylbenzenesulfonate (**19m**)

Following the general procedure, 2-(4-(trifluoromethoxy)-3-(trifluoromethyl)phenyl)ethanol **18m** (302 mg, 1.10 mmol), TEA (0.76 mL, 5.50 mmol) and TsCl (524 mg, 2.75 mmol) gave the desired compound **19m** (311 mg, 66%); ^{1}H NMR (400 MHz, $CDCl_3$) δ 7.68 (d, J = 8.3 Hz, 2H), 7.42–7.38 (m, 2H), 7.33–7.29 (m, 3H), 4.28 (t, J = 6.4 Hz, 2H), 3.04 (t, J = 6.4 Hz, 2H), 2.46 (s, 3H).

1.7.14. 4-Methyl-3-(trifluoromethyl)phenethyl 4-methylbenzenesulfonate (**19n**)

Following the general procedure, 2-(4-methyl-3-(trifluoromethyl)phenyl)ethanol **18n** (448 mg, 2.19 mmol), TEA (1.52 mL, 10.95 mmol) and TsCl (1.05 g, 5.49 mmol) gave the desired compound **19n** (503 mg, 64%); ^{1}H NMR (400 MHz, $CDCl_3$) δ 7.69 (d, J = 8.3 Hz, 2H), 7.31–7.28 (m, 3H), 7.23–7.18 (m, 2H), 4.24 (t, J = 6.7 Hz, 2H), 2.98 (t, J = 6.7 Hz, 2H), 2.47–2.46 (m, 6H).

1.7.15. 2-Methyl-5-(trifluoromethyl)phenethyl 4-methylbenzenesulfonate (**19o**)

Following the general procedure, 2-(2-methyl-5-(trifluoromethyl)phenyl)ethanol **18o** (174 mg, 0.85 mmol), TEA (0.59 mL, 4.25 mmol) and TsCl (324 mg, 1.70 mmol) gave the desired compound **19o** (261 mg, 85%); ^{1}H NMR (400 MHz, $CDCl_3$) δ 7.67 (d, J = 8.0 Hz, 2H), 7.38 (d, J = 7.6 Hz, 1H), 7.30–7.22 (m, 4H), 4.24 (t, J = 5.8 Hz, 2H), 3.03 (t, J = 7.0 Hz, 2H), 2.44 (s, 3H), 2.32 (s, 3H).

1.7.16. 5-Methyl-2-(trifluoromethyl)phenethyl 4-methylbenzenesulfonate (**19p**)

Following the general procedure, 2-(5-methyl-2-(trifluoromethyl)phenyl)ethanol **18p** (277 mg, 1.36 mmol), TEA (1.13 mL, 8.16 mmol) and TsCl (776 mg, 4.07 mmol) gave the desired compound **19p** (364 mg, 75%); ^{1}H NMR (400 MHz, $CDCl_3$) δ 7.74 (d, J = 8.3 Hz, 2H), 7.50 (d, J = 8.0 Hz, 1H), 7.32 (d, J = 8.0 Hz, 2H), 7.15 (d, J = 8.6 Hz, 1H), 7.12 (s, 1H) 4.24 (t, J = 7.0 Hz, 2H), 3.13 (t, J = 7.0 Hz, 2H), 2.46 (s, 3H), 2.38 (s, 3H).

2. Spectra of T-type calcium channel inhibitors

^{1}H NMR spectrum of (*S*)-5-Isobutyl-1-phenyl-*N*-(pyrrolidin-3-ylmethyl)-1*H*-pyrazole-3-carboxamide (15)

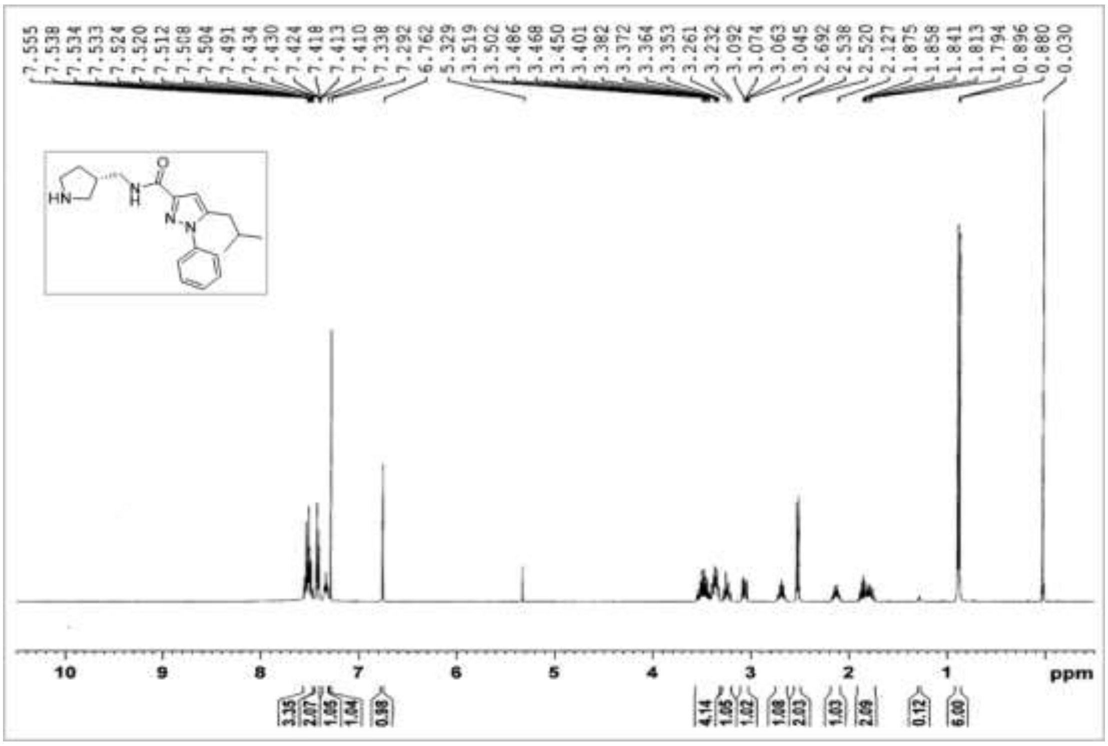

^{13}C NMR spectrum of (*S*)-5-Isobutyl-1-phenyl-*N*-(pyrrolidin-3-ylmethyl)-1*H*-pyrazole-3-carboxamide (15)

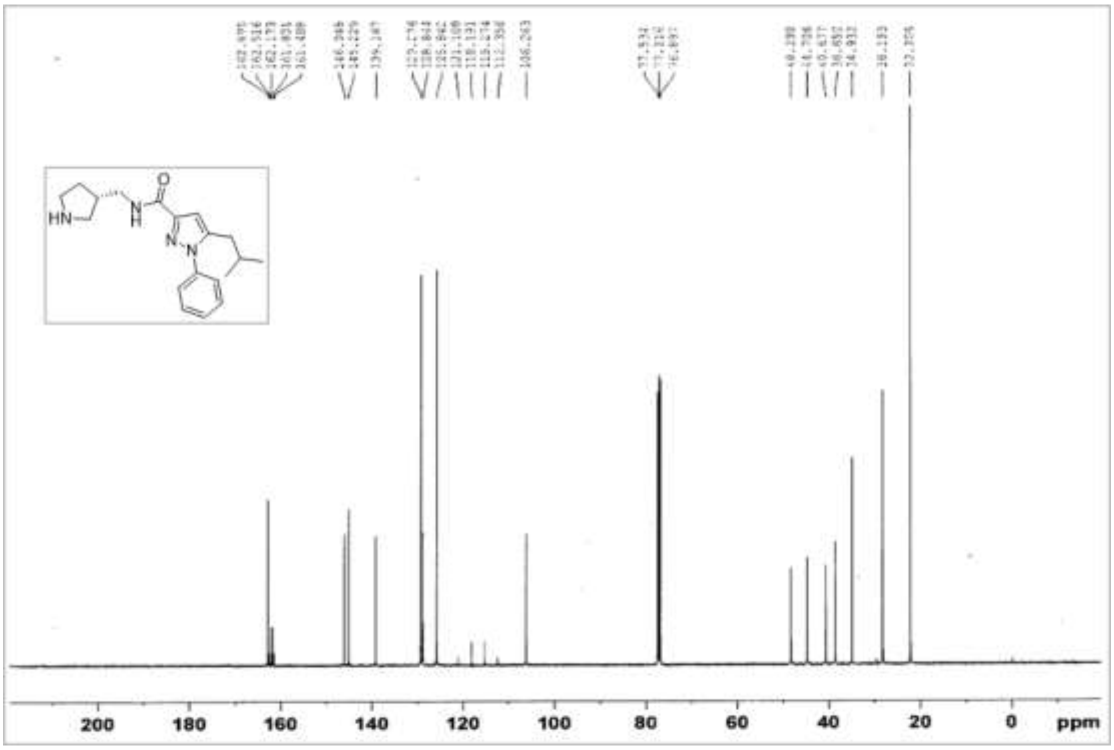

^{1}H NMR spectrum of (*R*)-*N*-((1-(3,3-dimethylbutyl)pyrrolidin-3-yl)methyl)-5-isobutyl- 1-phenyl-1*H*-pyrazole -3-carboxamide (16)

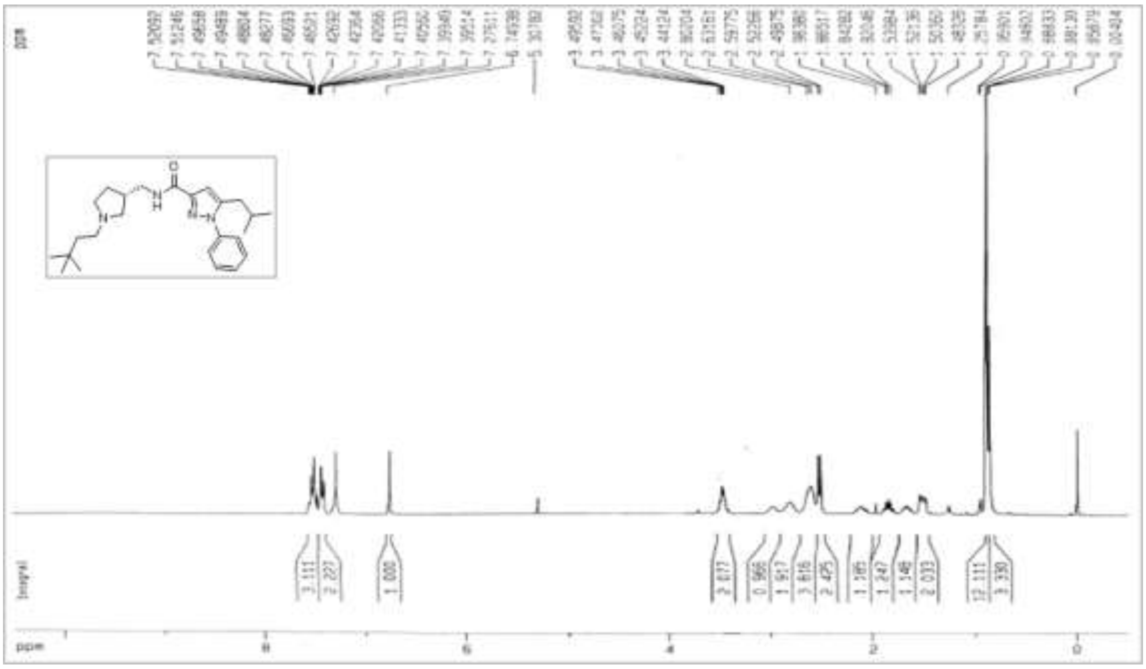

^{13}C NMR spectrum of (*R*)-*N*-((1-(3,3-dimethylbutyl)pyrrolidin-3-yl)methyl)-5-isobutyl- 1-phenyl-1*H*-pyrazole -3-carboxamide (16)

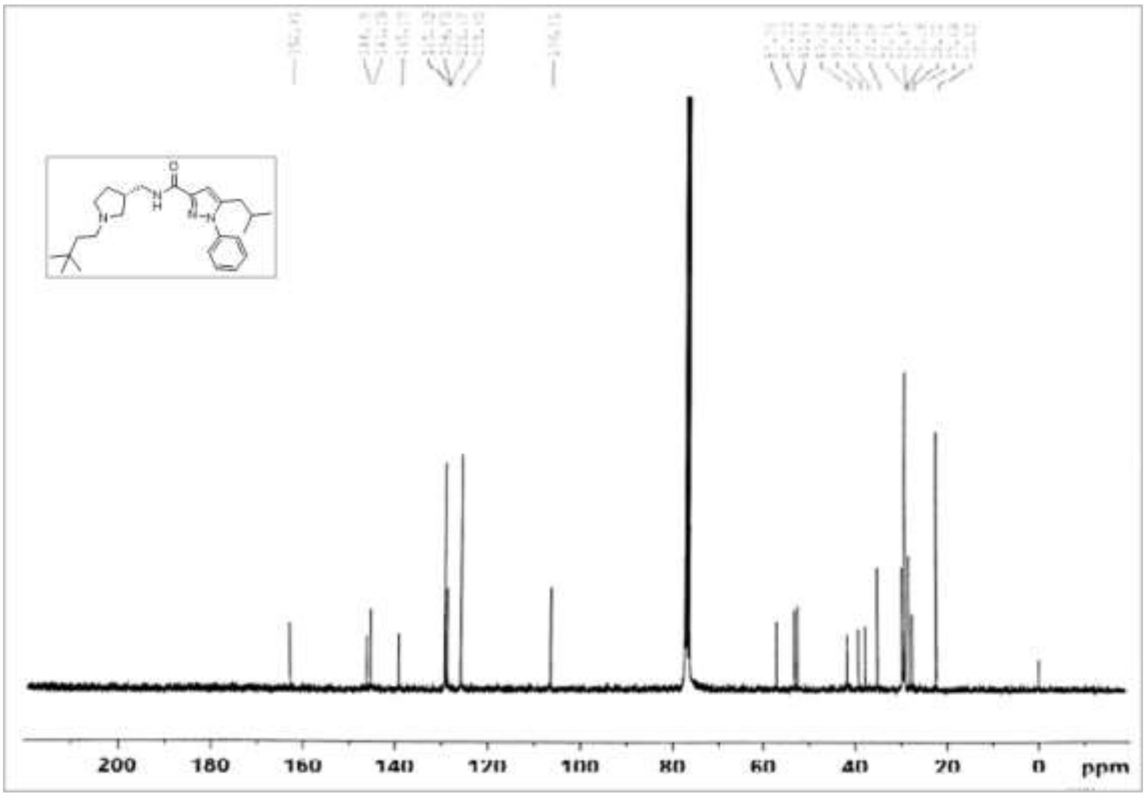

^{1}H NMR spectrum of (*R*)-5-isobutyl-1-phenyl-*N*-((1-(4-(trifluoromethyl)phenethyl)pyrrolidin-3-yl)methyl)-1*H*-pyrazole-3-carboxamide (20a)

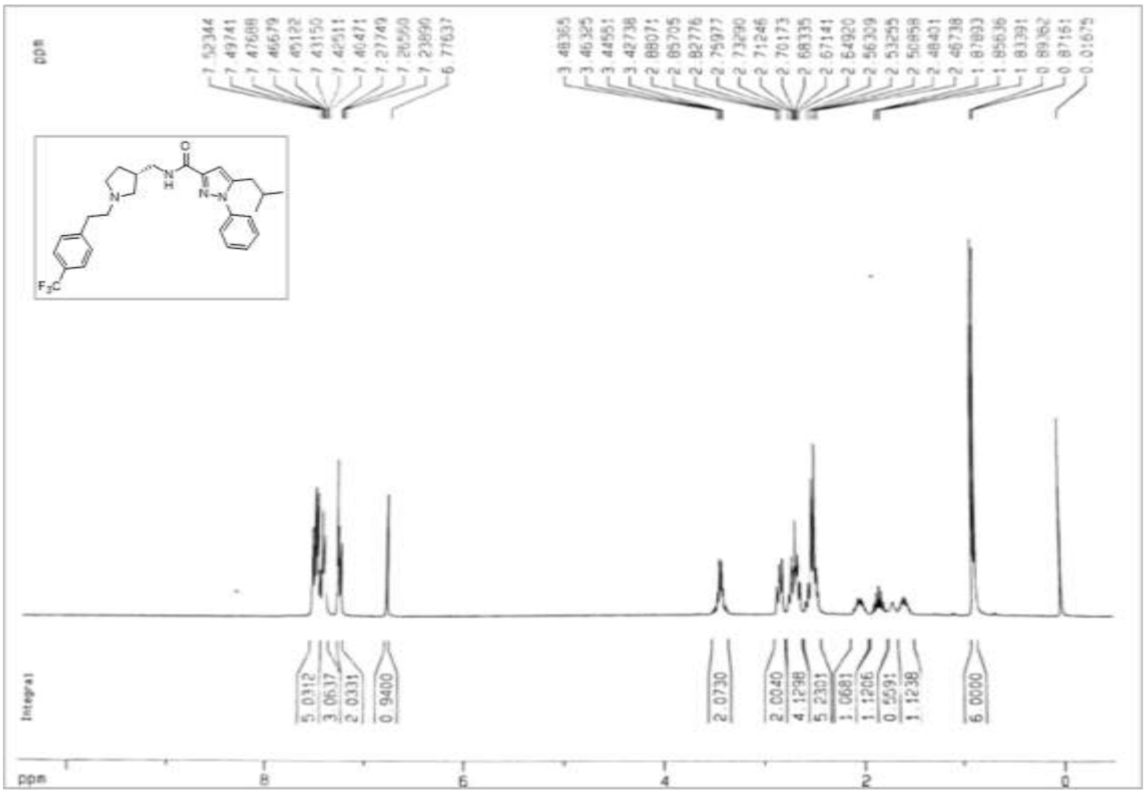

^{13}C NMR spectrum of (*R*)-5-isobutyl-1-phenyl-*N*-((1-(4-(trifluoromethyl)phenethyl)pyrrolidin-3-yl)methyl)-1*H*-pyrazole-3-carboxamide (20a)

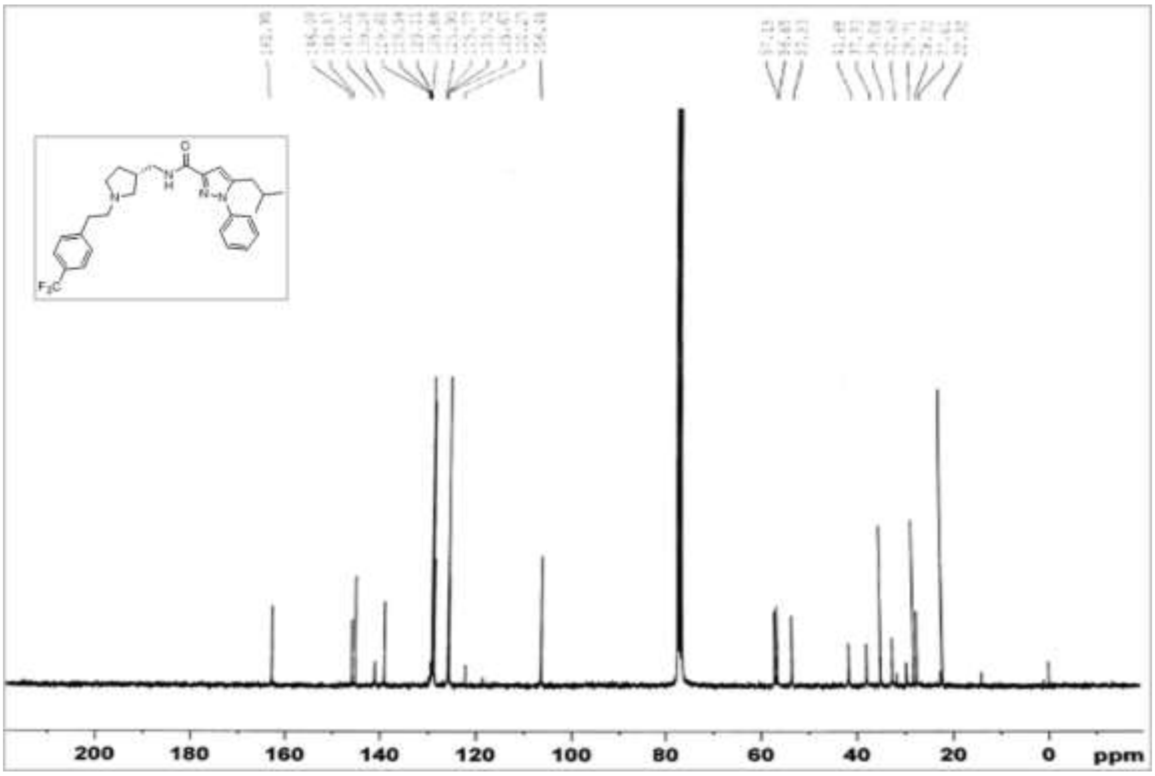

^{1}H NMR spectrum of (*R*)-5-isobutyl-1-phenyl-*N*-((1-(3-(trifluoromethyl)phenethyl)pyrrolidin-3-yl)methyl)-1*H*-pyrazole-3-carboxamide (20b)

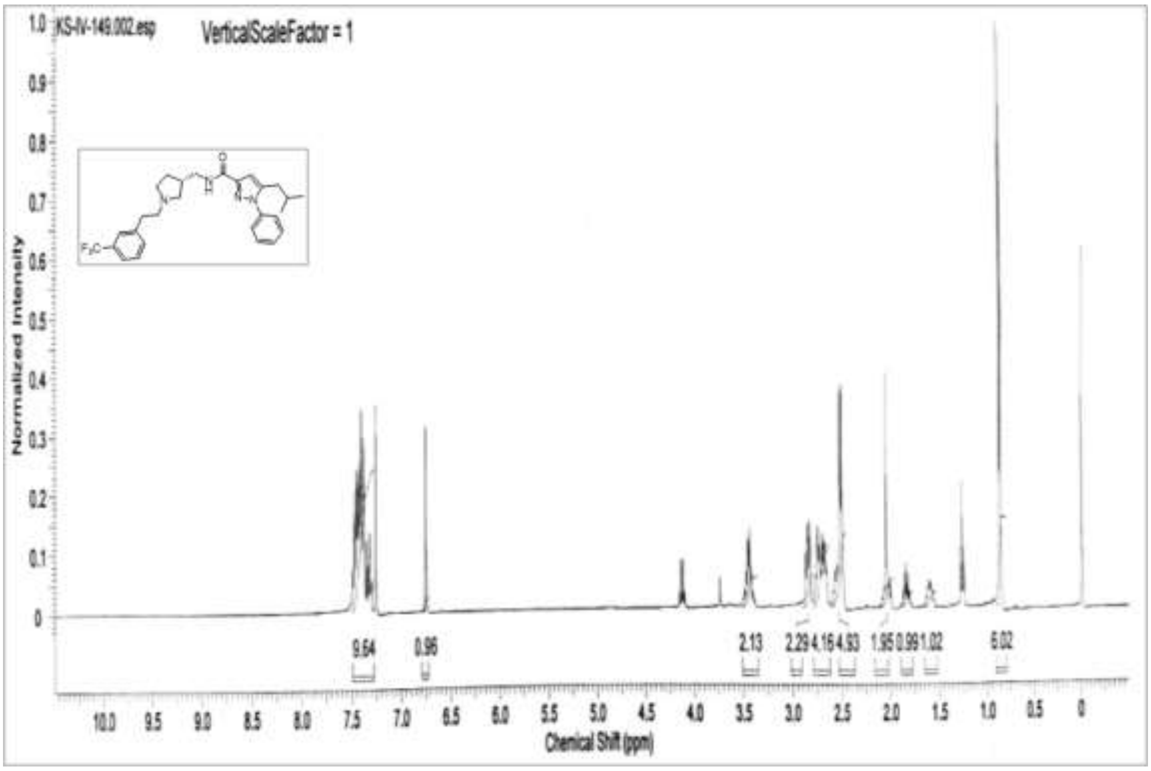

^{13}C NMR spectrum of (*R*)-5-isobutyl-1-phenyl-*N*-((1-(3-(trifluoromethyl)phenethyl)pyrrolidin-3-yl)methyl)-1*H*-pyrazole-3-carboxamide (20b)

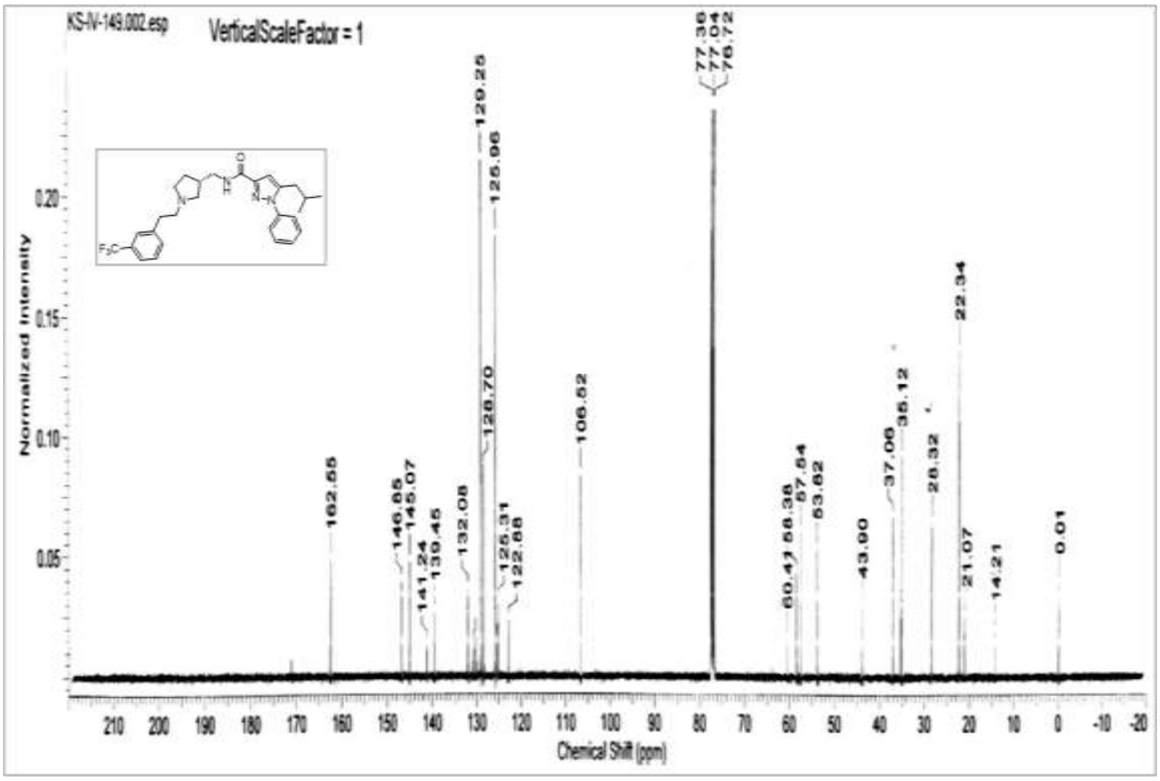

^{1}H NMR spectrum of (*R*)-5-isobutyl-1-phenyl-*N*-((1-(2-(trifluoromethyl)phenethyl)pyrrolidin-3-yl)methyl)-1*H*-pyrazole-3-carboxamide (20c)

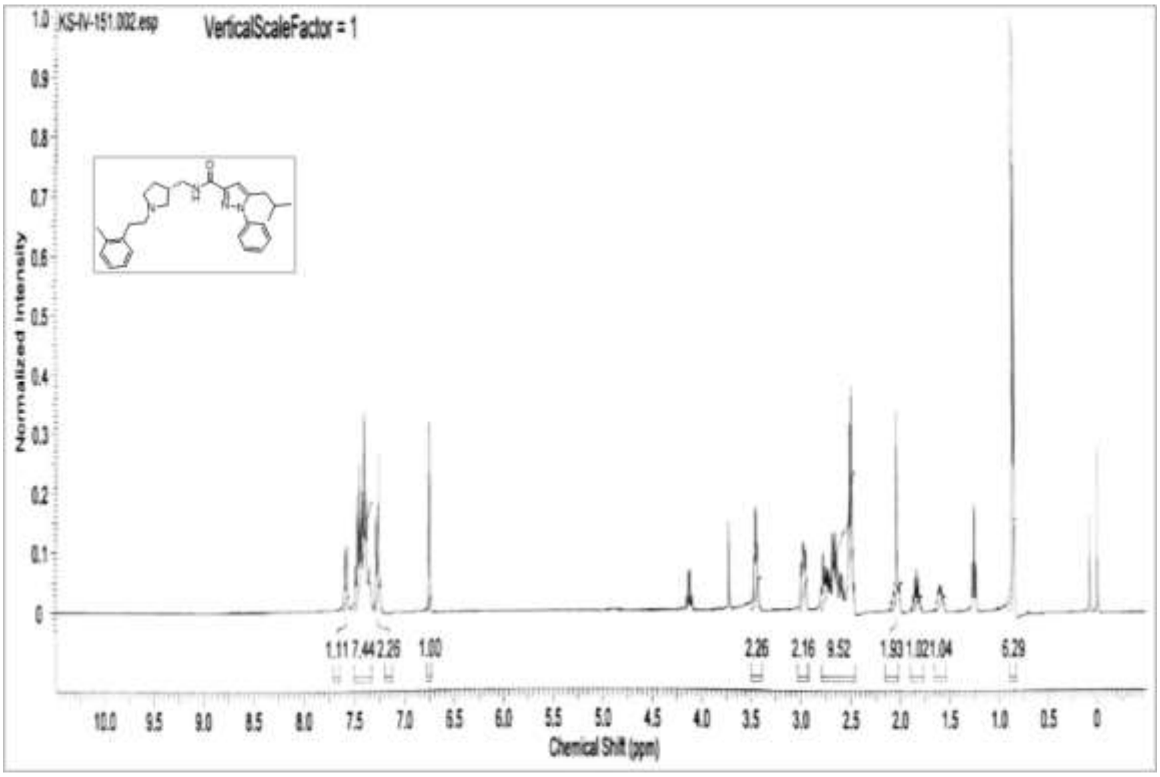

^{13}C NMR spectrum of (*R*)-5-isobutyl-1-phenyl-*N*-((1-(2-(trifluoromethyl)phenethyl)pyrrolidin-3-yl)methyl)-1*H*-pyrazole-3-carboxamide (20c)

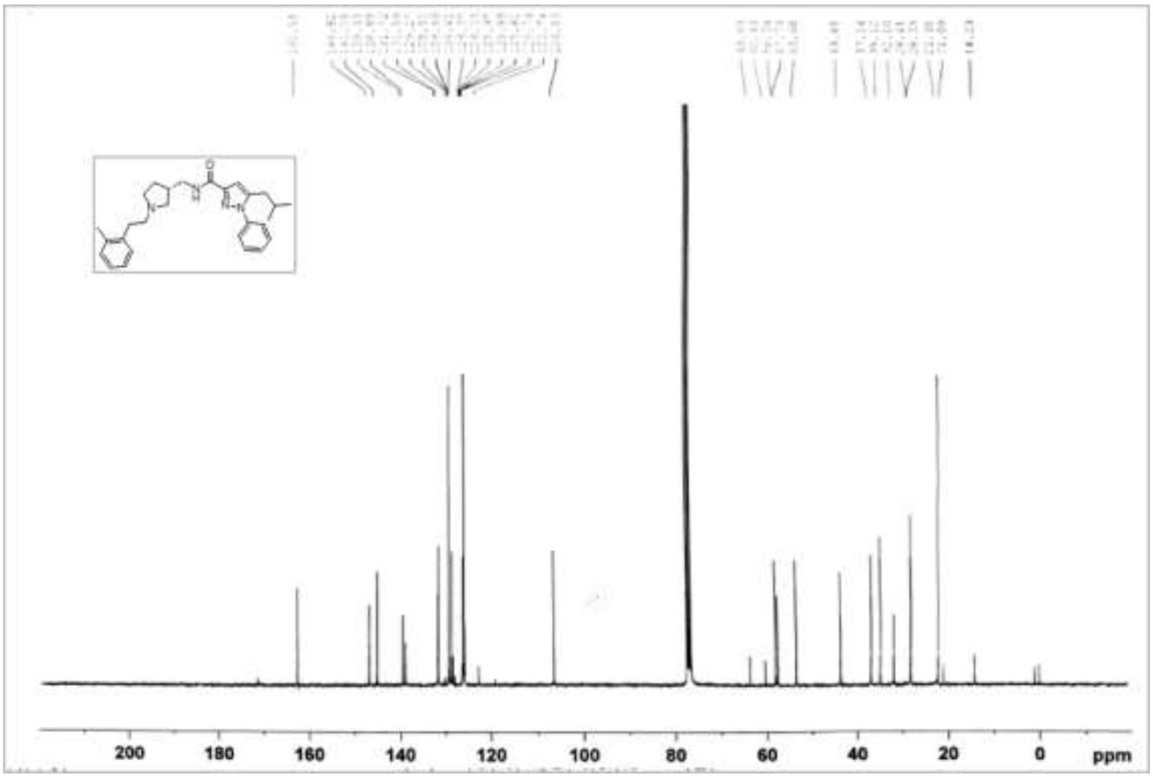

^{1}H NMR spectrum of (*R*)-*N*-((1-(3-fluorophenethyl)pyrrolidin-3-yl)methyl)-5-isobutyl-1-phenyl-1*H*-pyrazole-3-carboxamide (20d)

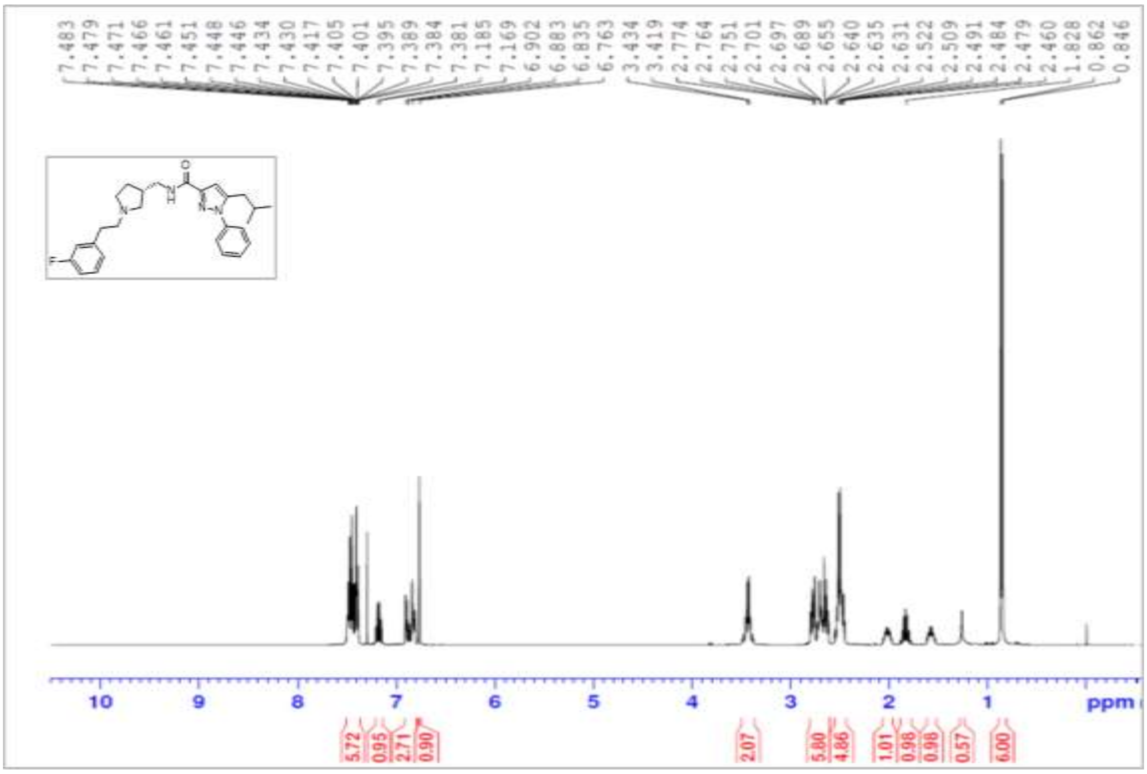

^{13}C NMR spectrum of (*R*)-*N*-((1-(3-fluorophenethyl)pyrrolidin-3-yl)methyl)-5-isobutyl-1-phenyl-1*H*-pyrazole-3-carboxamide (20d)

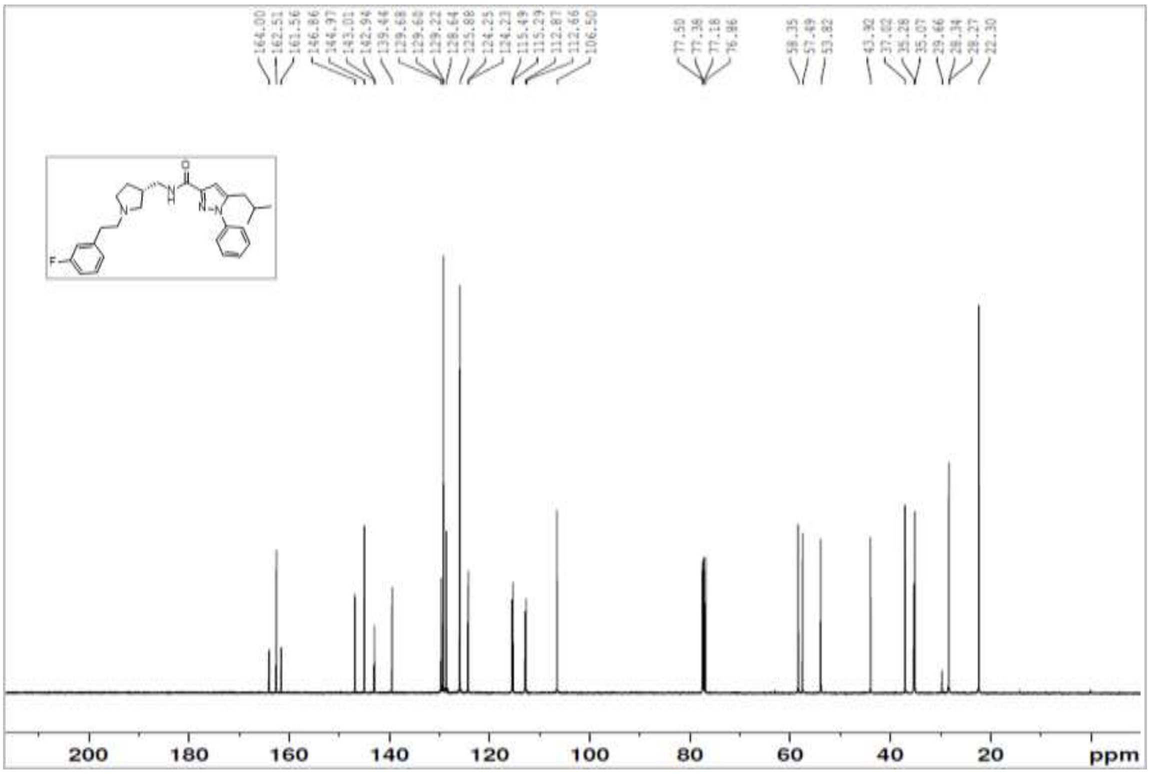

^{1}H NMR spectrum of (*R*)-*N*-((1-(3,4-difluorophenethyl)pyrrolidin-3-yl)methyl)-5-isobutyl-1-phenyl-1*H*-pyrazole-3-carboxamide (20e)

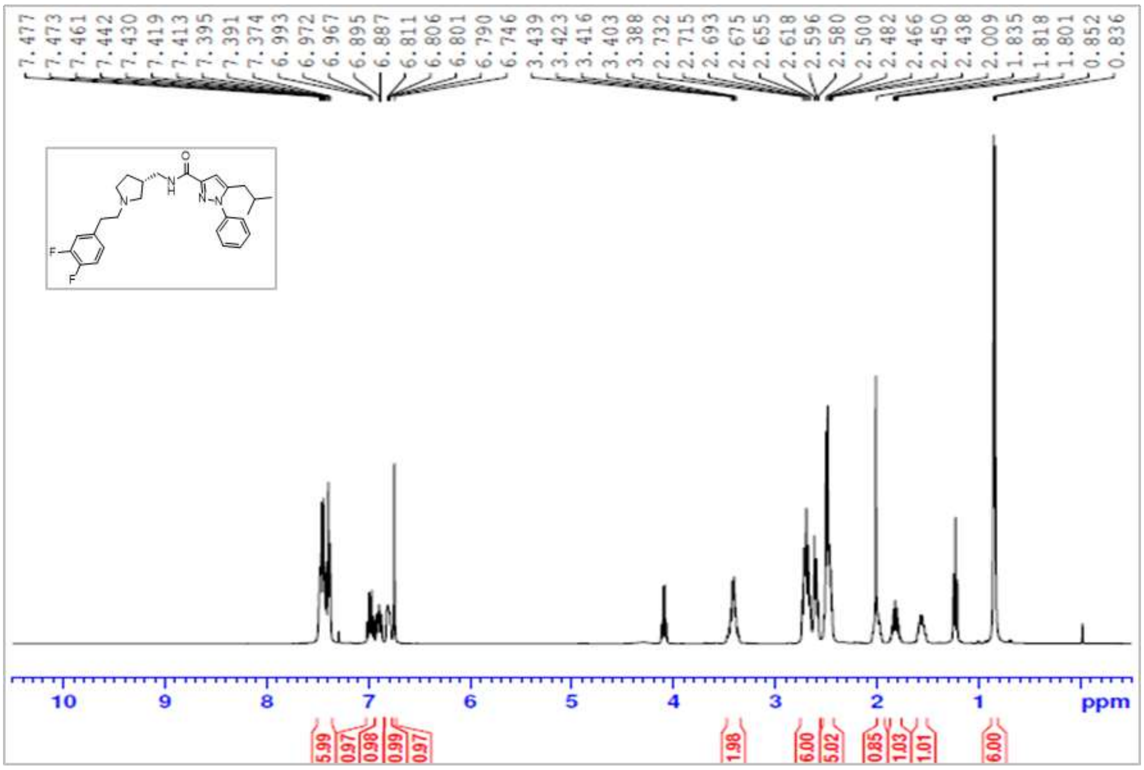

^{13}C NMR spectrum of (*R*)-*N*-((1-(3,4-difluorophenethyl)pyrrolidin-3-yl)methyl)-5-isobutyl-1-phenyl-1*H*-pyrazole-3-carboxamide (20e)

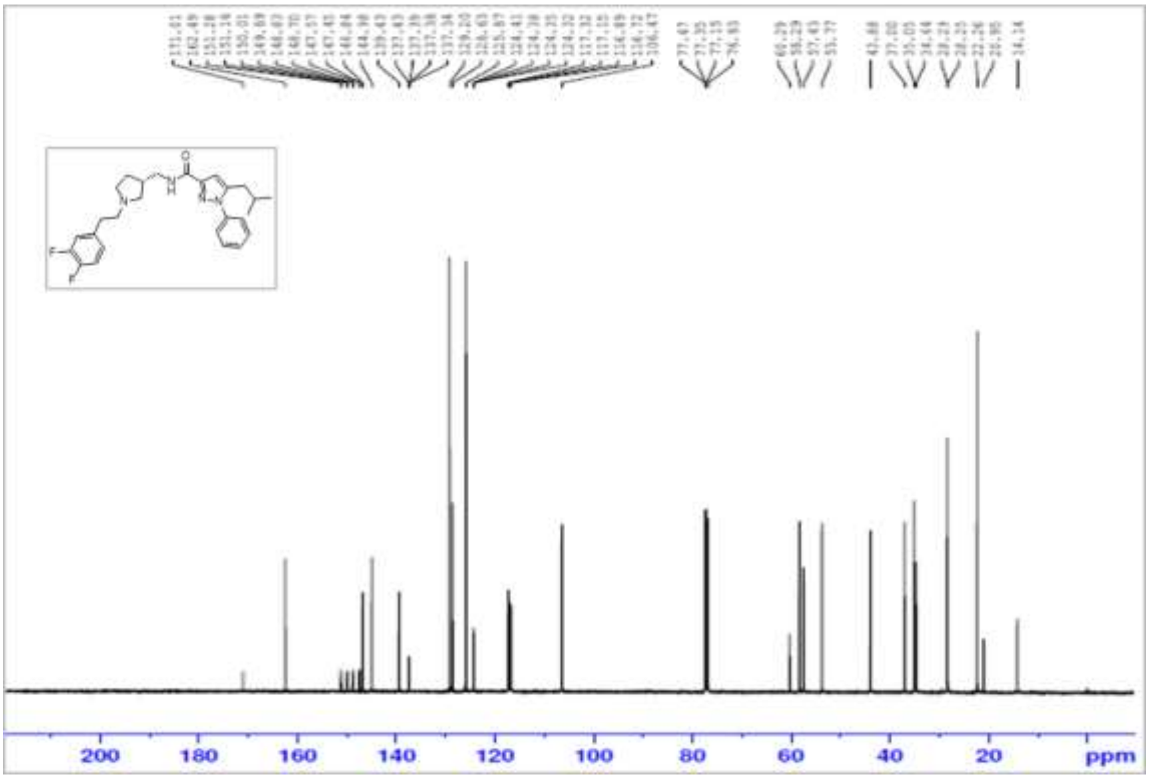

^{1}H NMR spectrum of (*R*)-*N*-((1-(3,5-difluorophenethyl)pyrrolidin-3-yl)methyl)-5-isobutyl-1-phenyl-1*H*-pyrazole-3-carboxamide (20f)

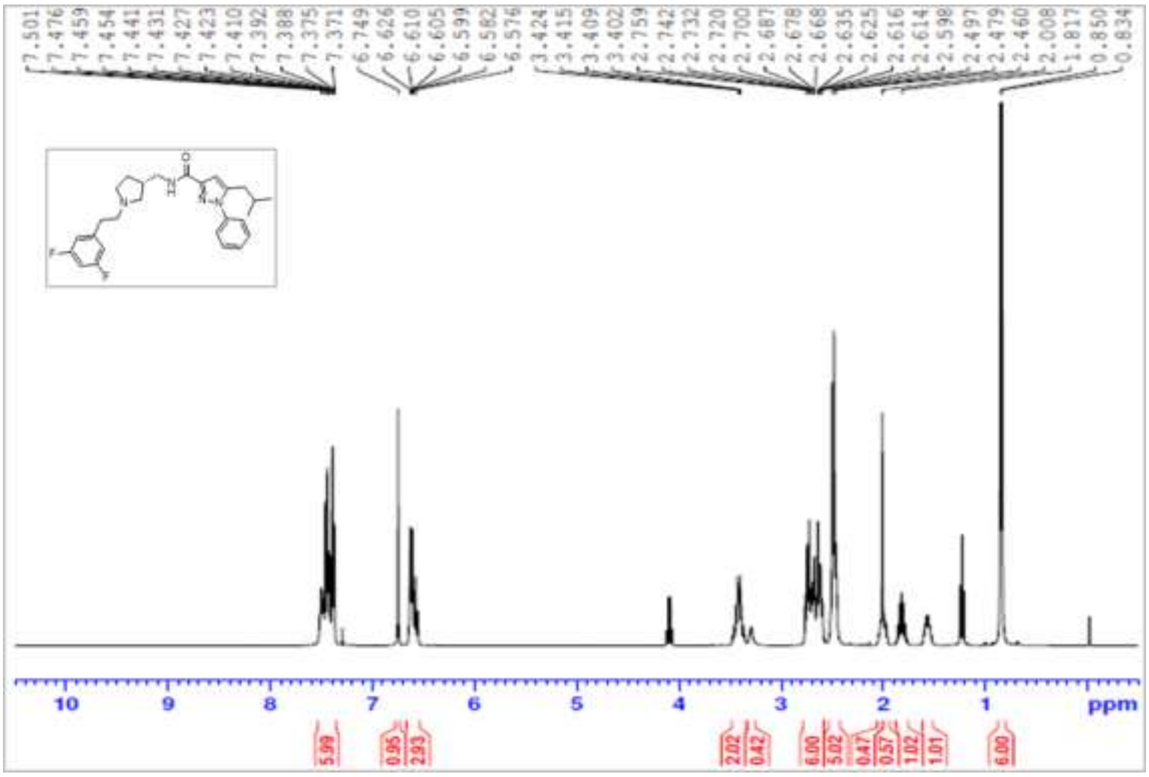

^{13}C NMR spectrum of (*R*)-*N*-((1-(3,5-difluorophenethyl)pyrrolidin-3-yl)methyl)-5-isobutyl-1-phenyl-1*H*-pyrazole-3-carboxamide (20f)

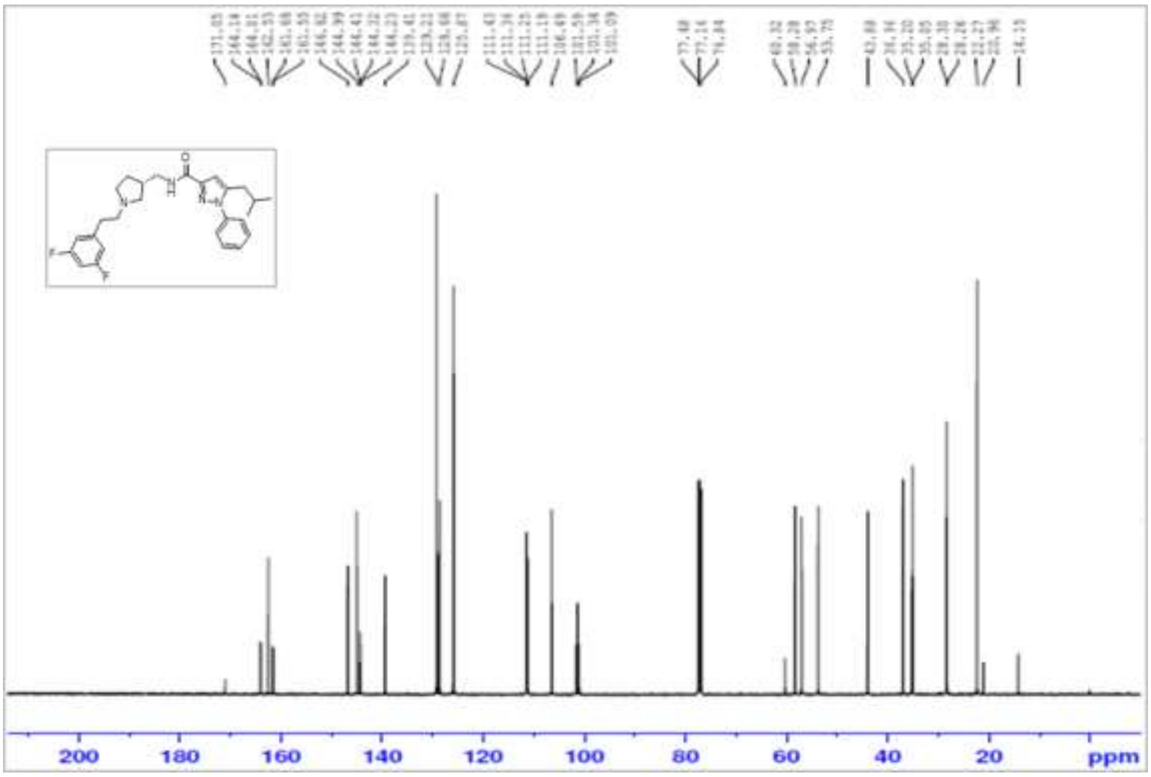

^{1}H NMR spectrum of (*R*)-*N*-((1-(2,3-difluorophenethyl)pyrrolidin-3-yl)methyl)-5-isobutyl-1-phenyl-1*H*-pyrazole-3-carboxamide (20g)

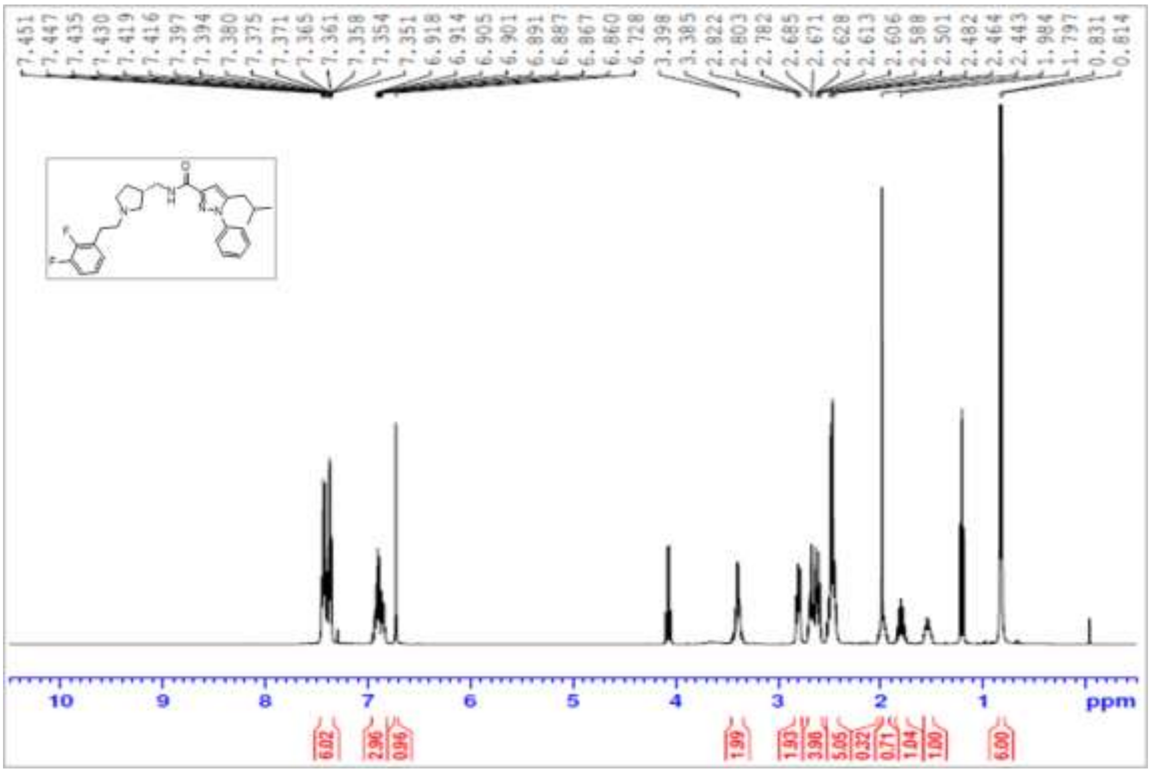

^{13}C NMR spectrum of (*R*)-*N*-((1-(2,3-difluorophenethyl)pyrrolidin-3-yl)methyl)-5-isobutyl-1-phenyl-1*H*-pyrazole-3-carboxamide (20g)

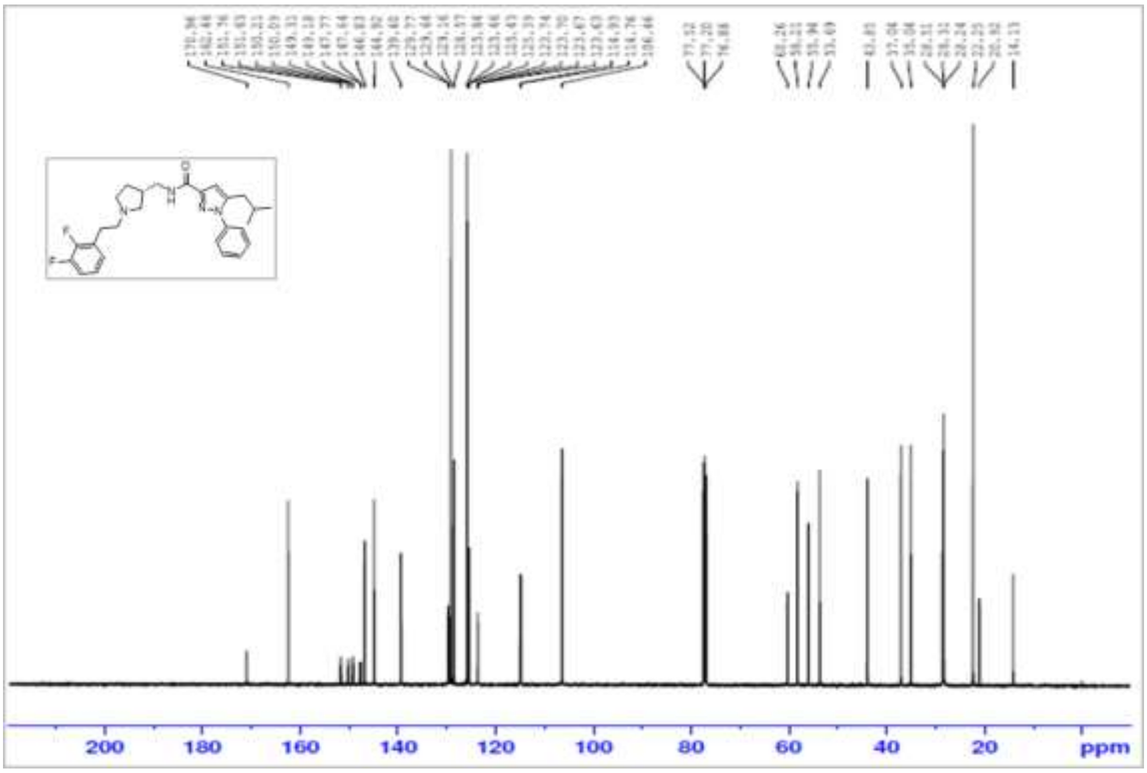

^{1}H NMR spectrum of (*R*)-*N*-((1-(3-chloro-5-fluorophenethyl)pyrrolidin-3-yl)methyl)-5-isobutyl-1-phenyl-1*H*-pyrazole-3-carboxamide (20h)

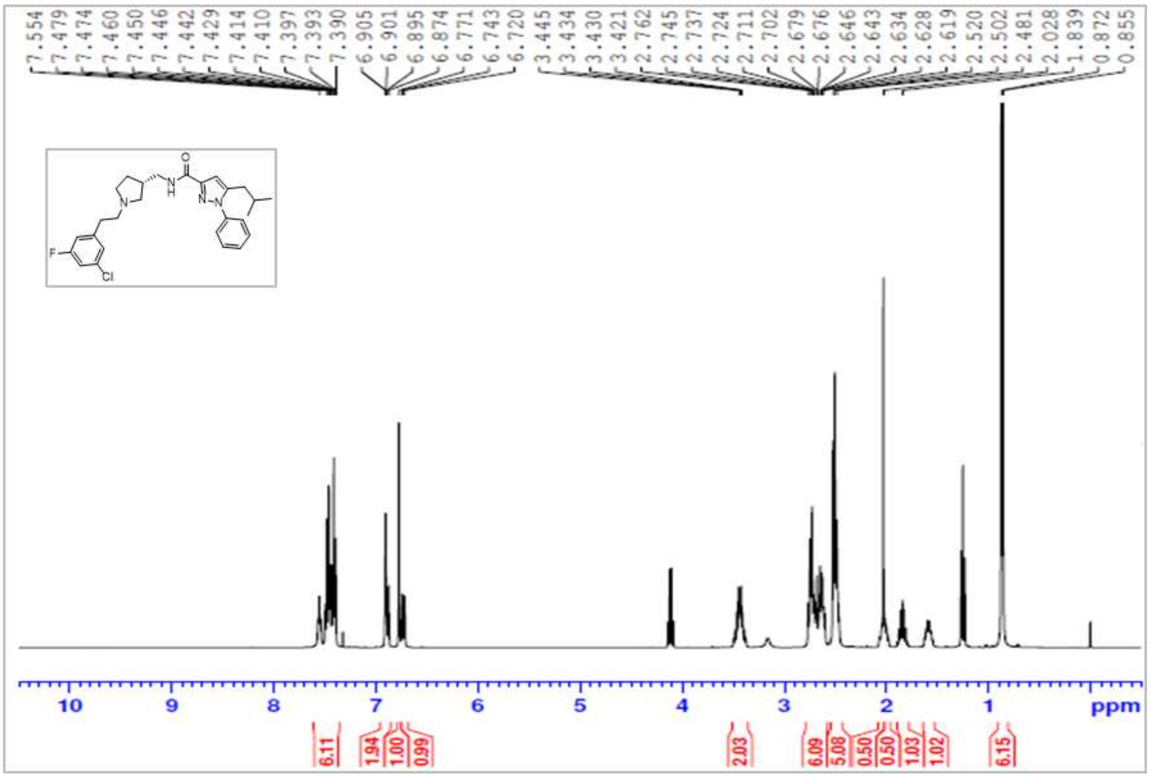

^{13}C NMR spectrum of (*R*)-*N*-((1-(3-chloro-5-fluorophenethyl)pyrrolidin-3-yl)methyl)-5-isobutyl-1-phenyl-1*H*-pyrazole-3-carboxamide (20h)

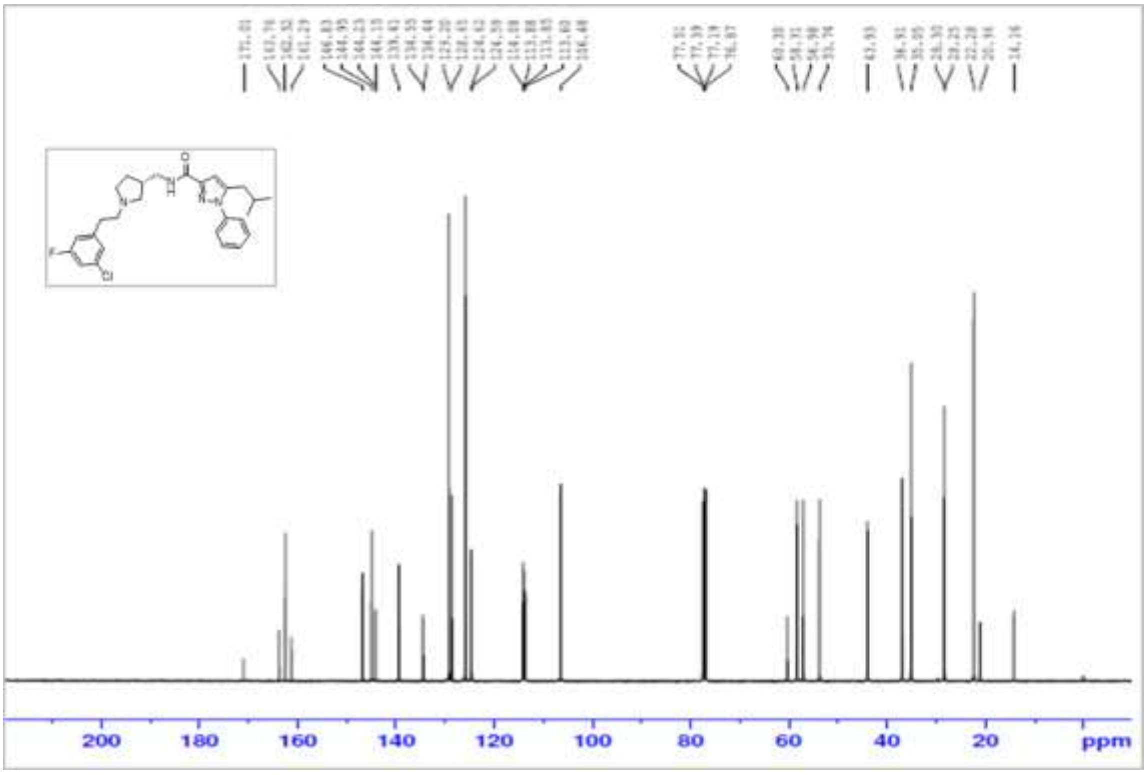

^{1}H NMR spectrum of (*R*)-*N*-((1-(4-chloro-3-fluorophenethyl)pyrrolidin-3-yl)methyl)-5-isobutyl-1-phenyl-1*H*-pyrazole-3-carboxamide (20i)

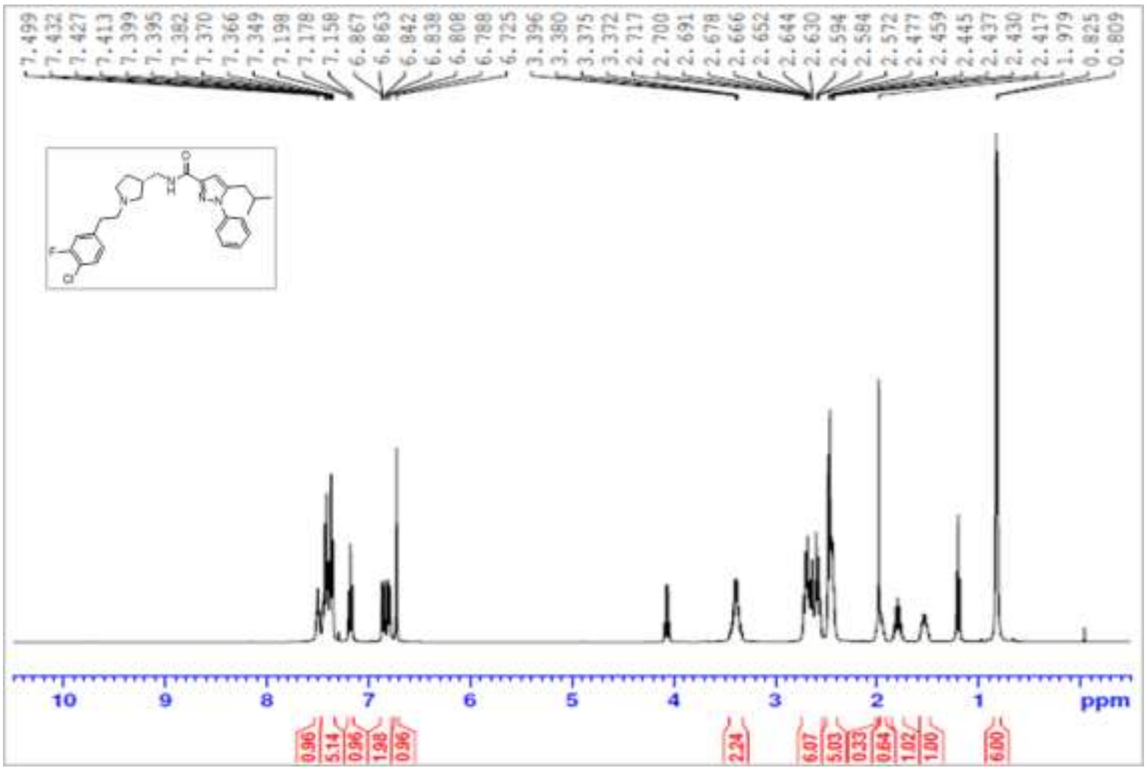

^{13}C NMR spectrum of (*R*)-*N*-((1-(4-chloro-3-fluorophenethyl)pyrrolidin-3-yl)methyl)-5-isobutyl-1-phenyl-1*H*-pyrazole-3-carboxamide (20i)

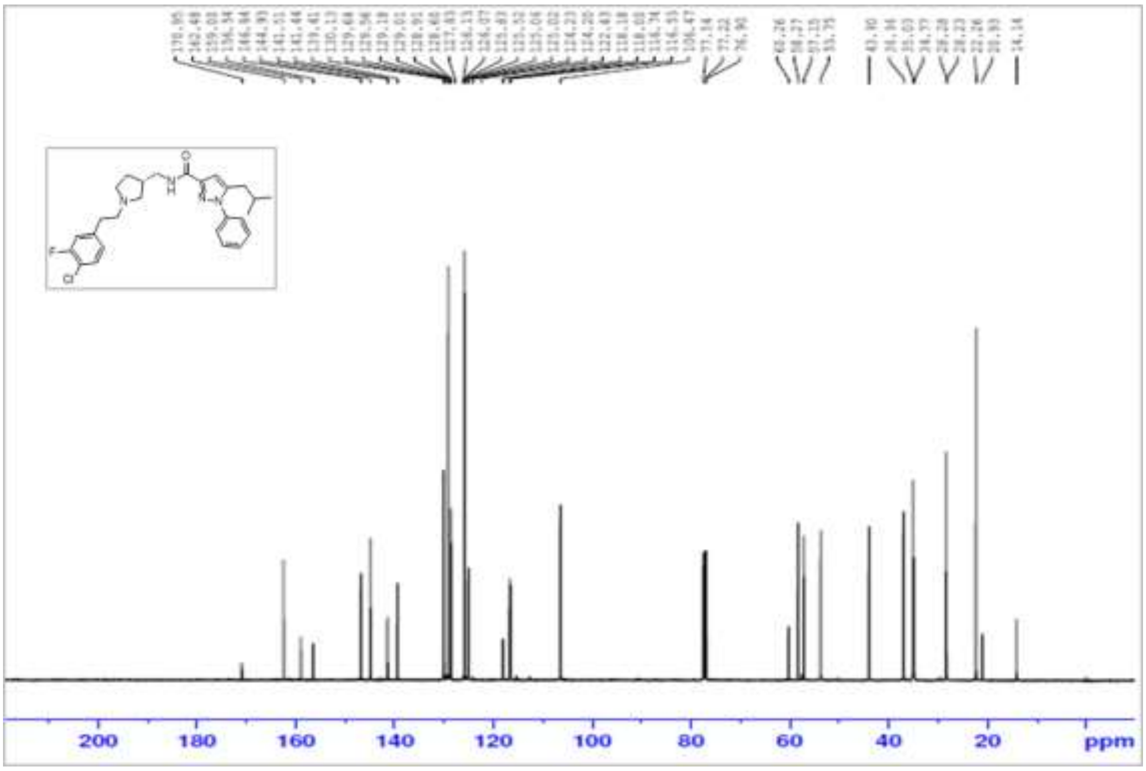

^{1}H NMR spectrum of (*R*)-*N*-((1-(3,4-dichlorophenethyl)pyrrolidin-3-yl)methyl)-5-isobutyl-1-phenyl-1*H*-pyrazole-3-carboxamide (20j)

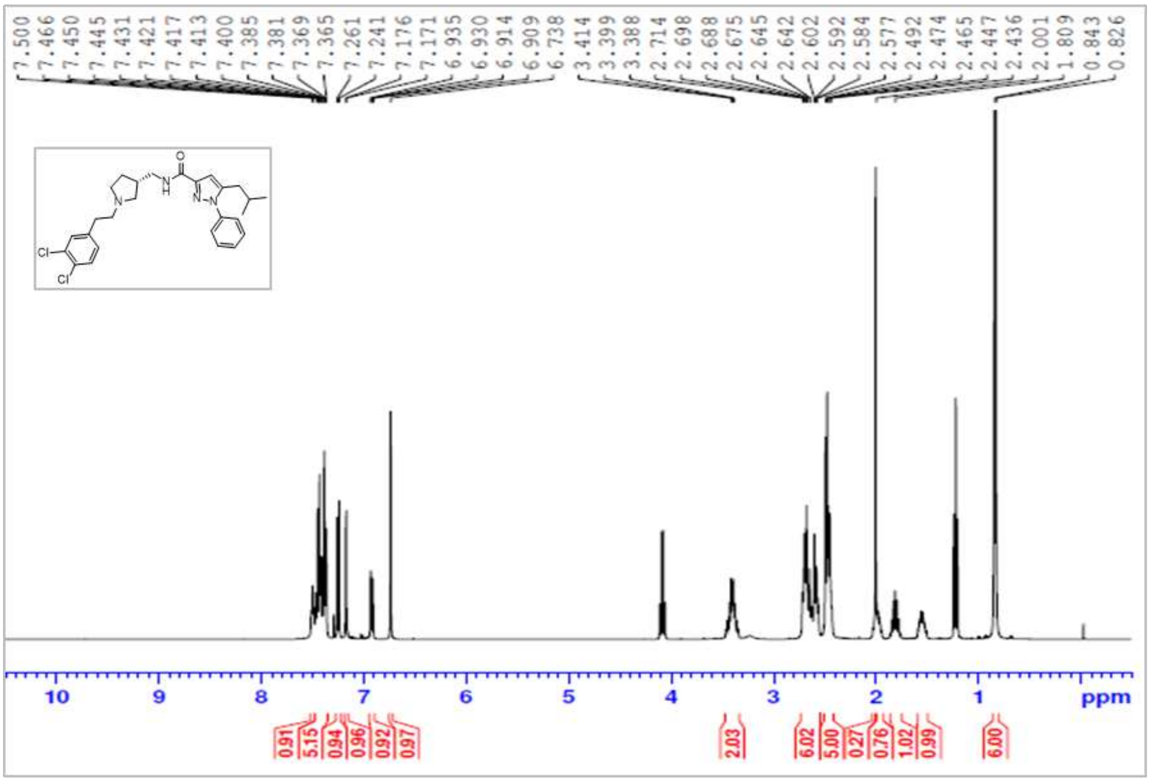

^{13}C NMR spectrum of (*R*)-*N*-((1-(3,4-dichlorophenethyl)pyrrolidin-3-yl)methyl)-5-isobutyl-1-phenyl-1*H*-pyrazole-3-carboxamide (20j)

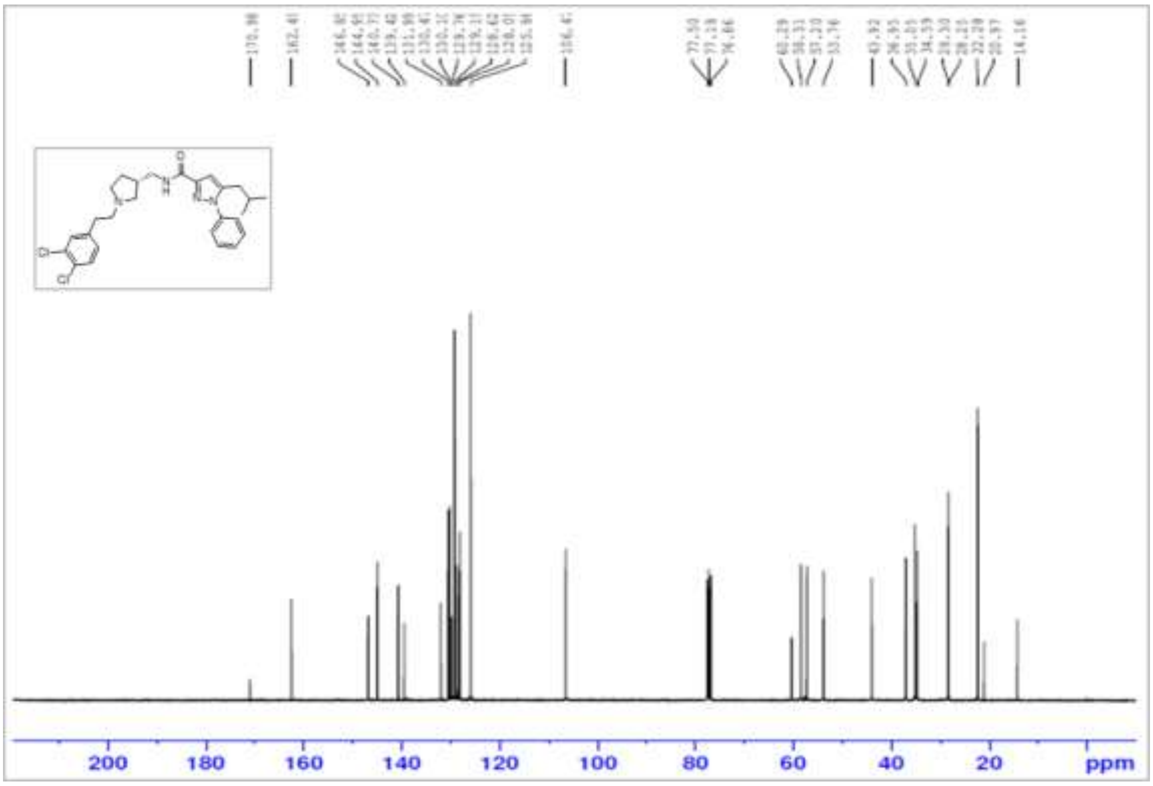

^{1}H NMR spectrum of (*R*)-*N*-((1-(2,4-bis(trifluoromethyl)phenethyl)pyrrolidin-3-yl)methyl)-5-isobutyl-1-phenyl-1*H*-pyrazole-3-carboxamide (20k)

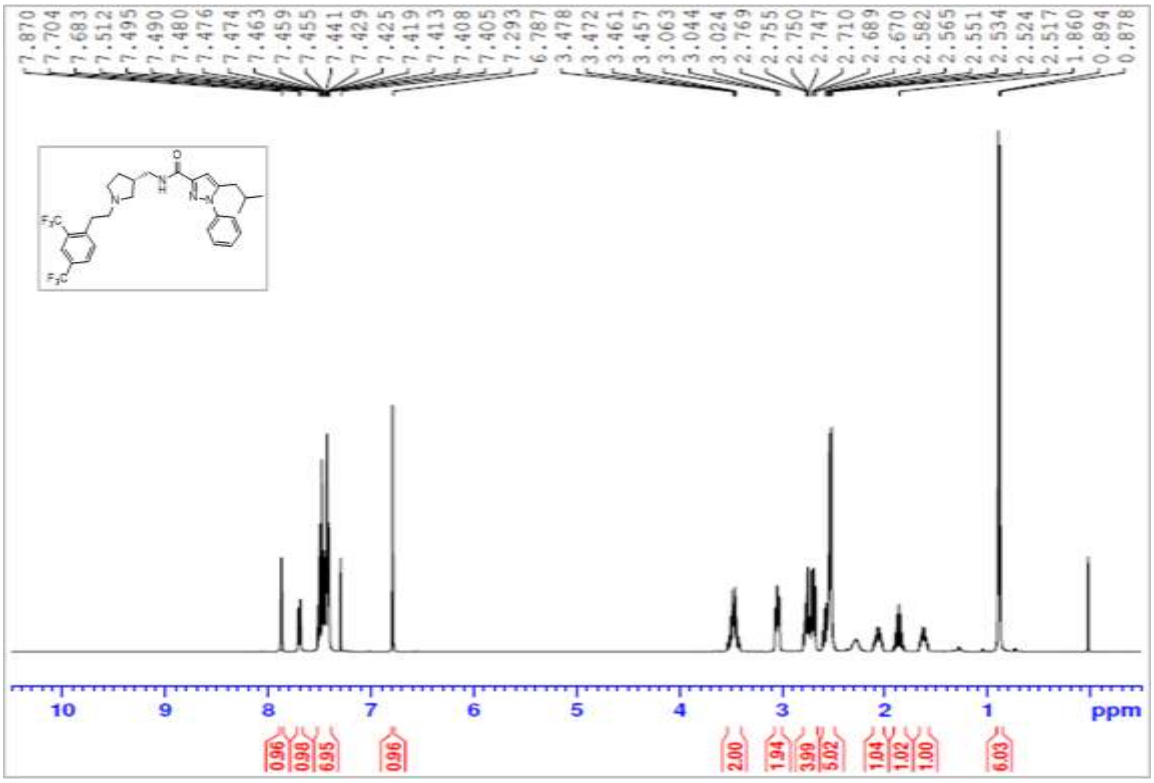

^{13}C NMR spectrum of (*R*)-*N*-((1-(2,4-bis(trifluoromethyl)phenethyl)pyrrolidin-3-yl)methyl)-5-isobutyl-1-phenyl-1*H*-pyrazole-3-carboxamide (20k)

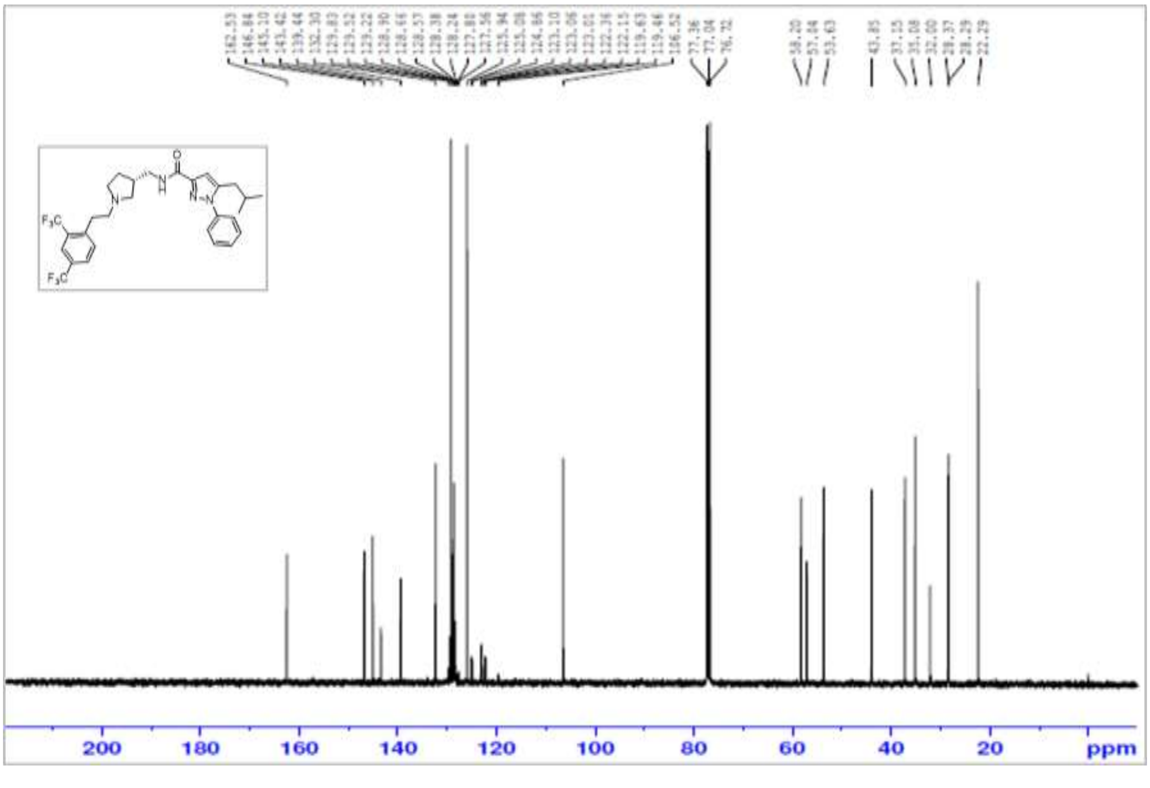

^{1}H NMR spectrum of (*R*)-*N*-((1-(3-fluoro-4-(trifluoromethoxy)phenethyl)pyrrolidin-3-yl)methyl)-5-isobutyl-1-phenyl-1*H*-pyrazole-3-carboxamide (20l)

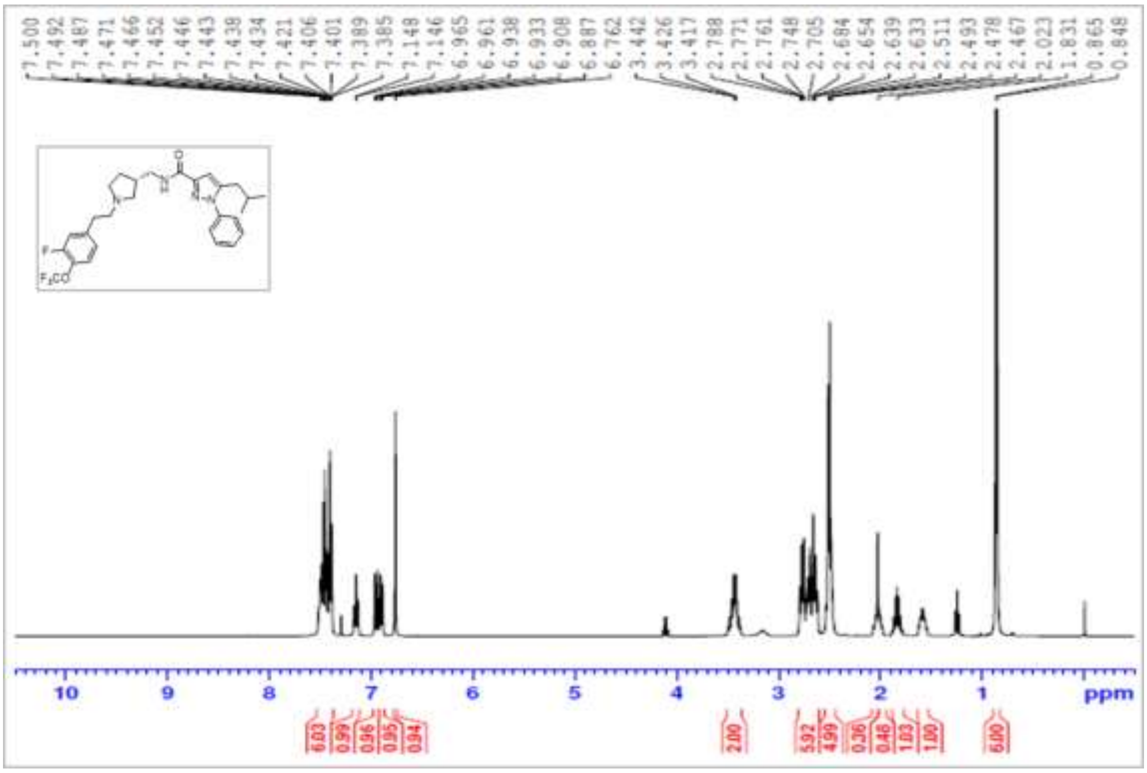

^{13}C NMR spectrum of (*R*)-*N*-((1-(3-fluoro-4-(trifluoromethoxy)phenethyl)pyrrolidin-3-yl)methyl)-5-isobutyl-1-phenyl-1*H*-pyrazole-3-carboxamide (20l)

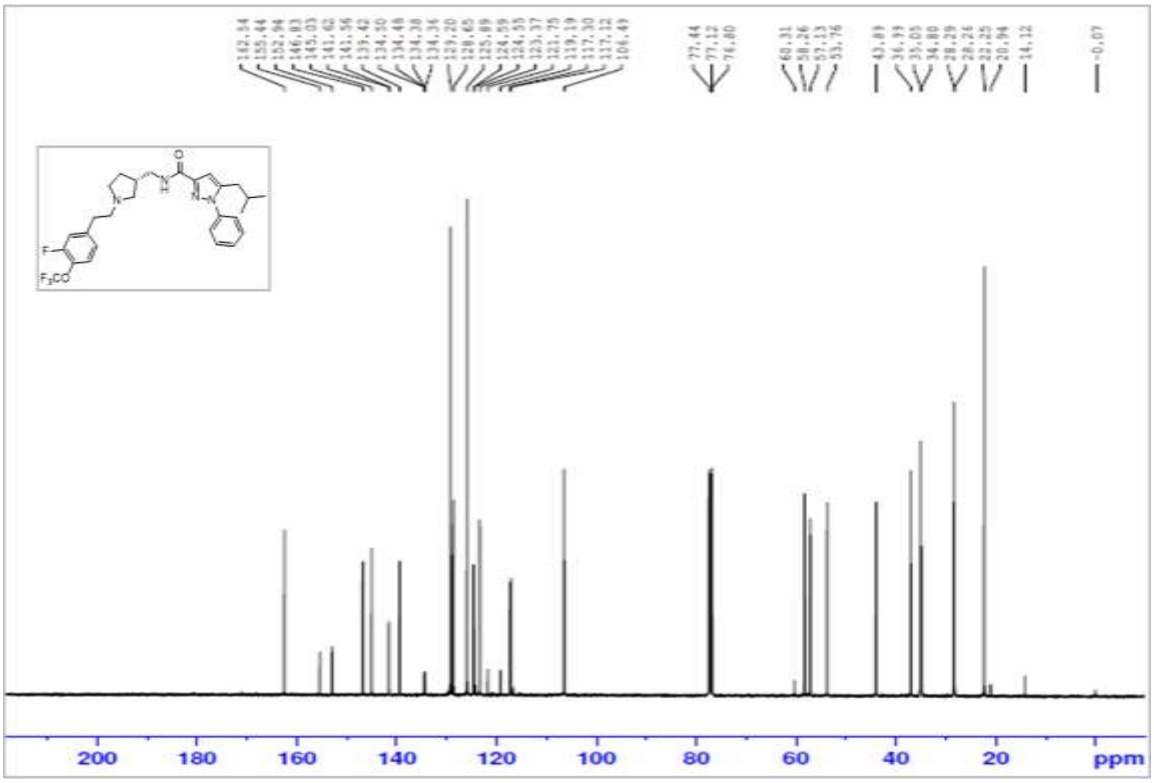

^{1}H NMR spectrum of (*R*)-5-isobutyl-1-phenyl-*N*-((1-(4-(trifluoromethoxy)-3-(trifluoromethyl)phenethyl)-pyrrolidin-3-yl)methyl)-1*H*-pyrazole-3-carboxamide (20m)

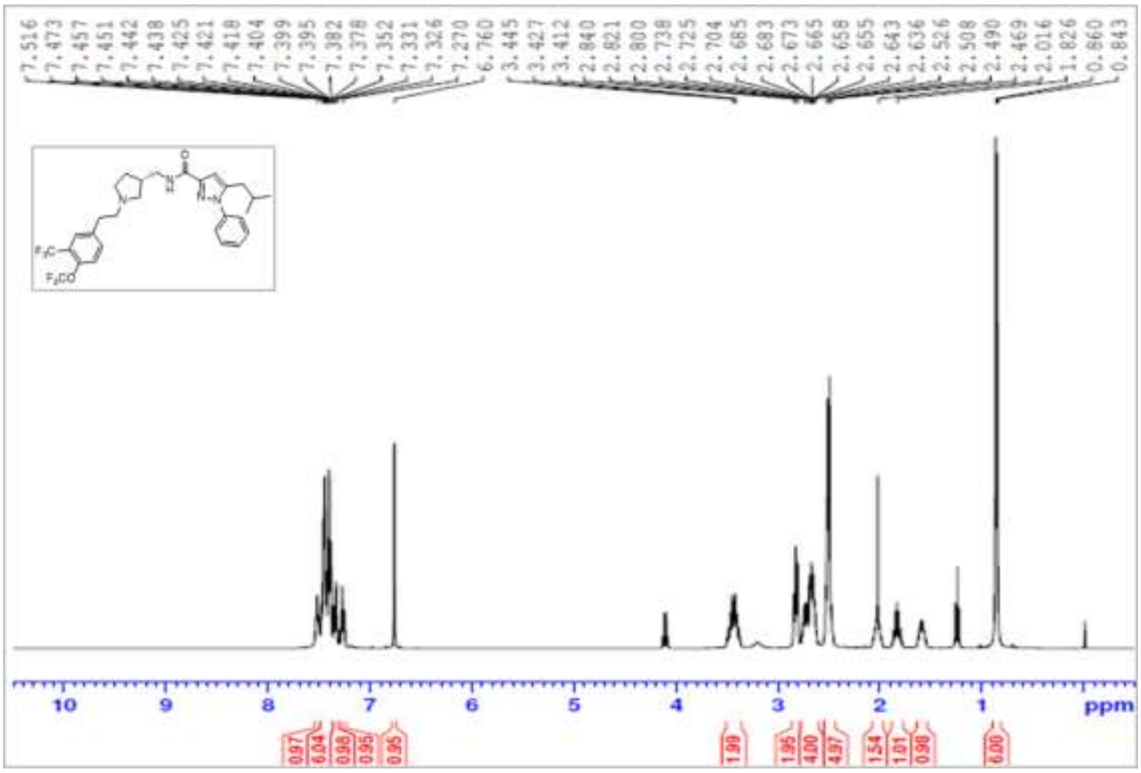

^{13}C NMR spectrum of (*R*)-5-isobutyl-1-phenyl-*N*-((1-(4-(trifluoromethoxy)-3-(trifluoromethyl)phenethyl)-pyrrolidin-3-yl)methyl)-1*H*-pyrazole-3-carboxamide (20m)

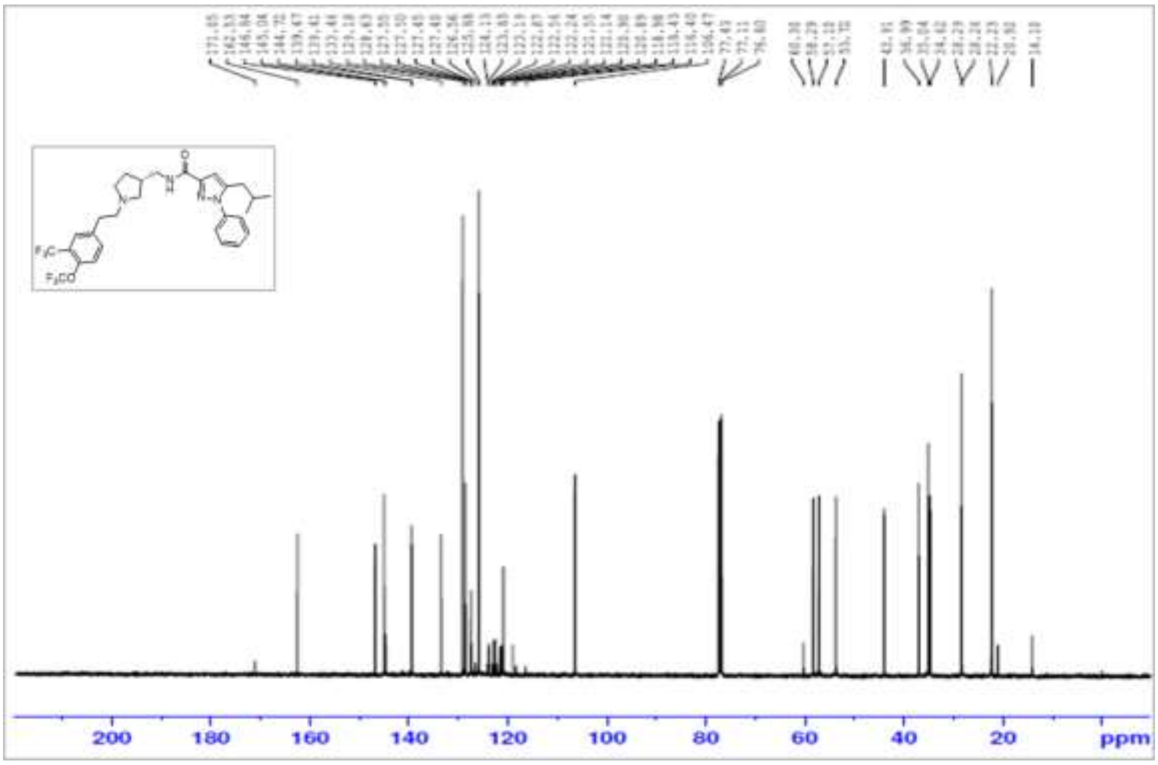

^{1}H NMR spectrum of (*R*)-5-Isobutyl-*N*-((1-(4-methyl-3-(trifluoromethyl)phenethyl)pyrrolidin-3-yl)methyl)-1-phenyl-1*H*-pyrazole-3-carboxamide (20n)

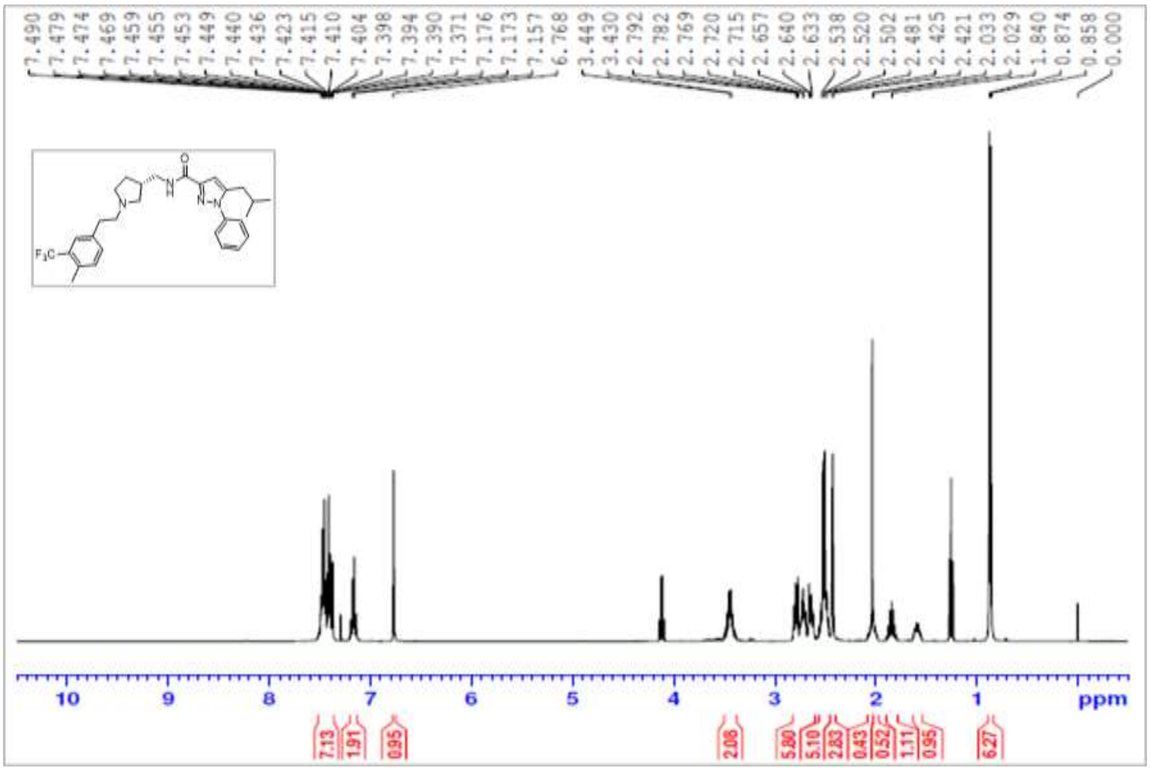

^{13}C NMR spectrum of (*R*)-5-Isobutyl-*N*-((1-(4-methyl-3-(trifluoromethyl)phenethyl)pyrrolidin-3-yl)methyl)-1-phenyl-1*H*-pyrazole-3-carboxamide (20n)

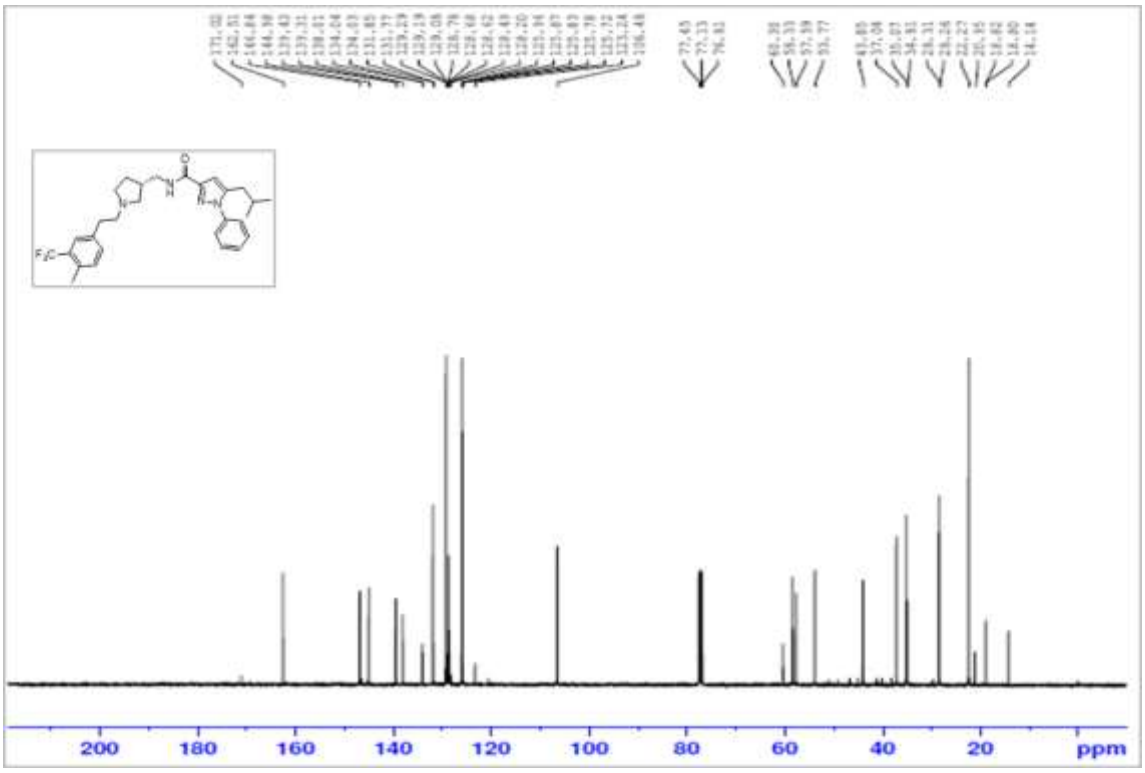

^{1}H NMR spectrum of (*R*)-5-Isobutyl-*N*-((1-(2-methyl-5-(trifluoromethyl)phenethyl)pyrrolidin-3-yl)methyl)-1-phenyl-1*H*-pyrazole-3-carboxamide (20o)

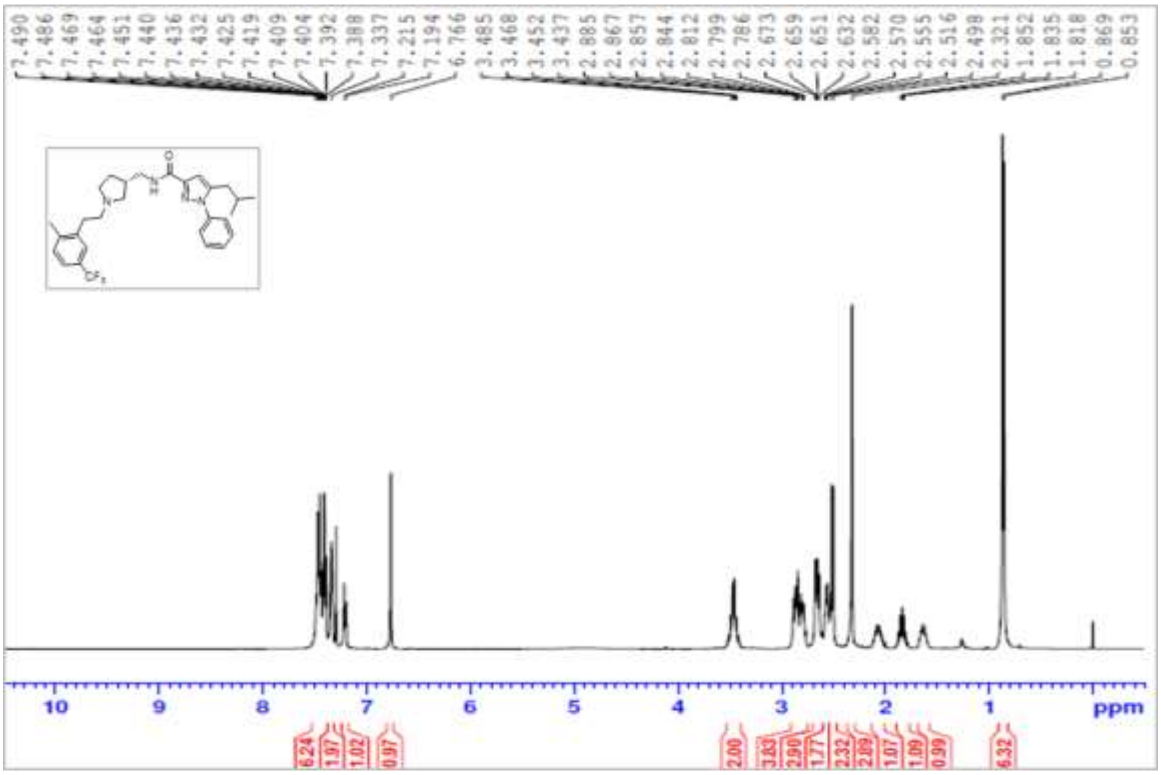

^{13}C NMR spectrum of (*R*)-5-Isobutyl-*N*-((1-(2-methyl-5-(trifluoromethyl)phenethyl)pyrrolidin-3-yl)methyl)-1-phenyl-1*H*-pyrazole-3-carboxamide (20o)

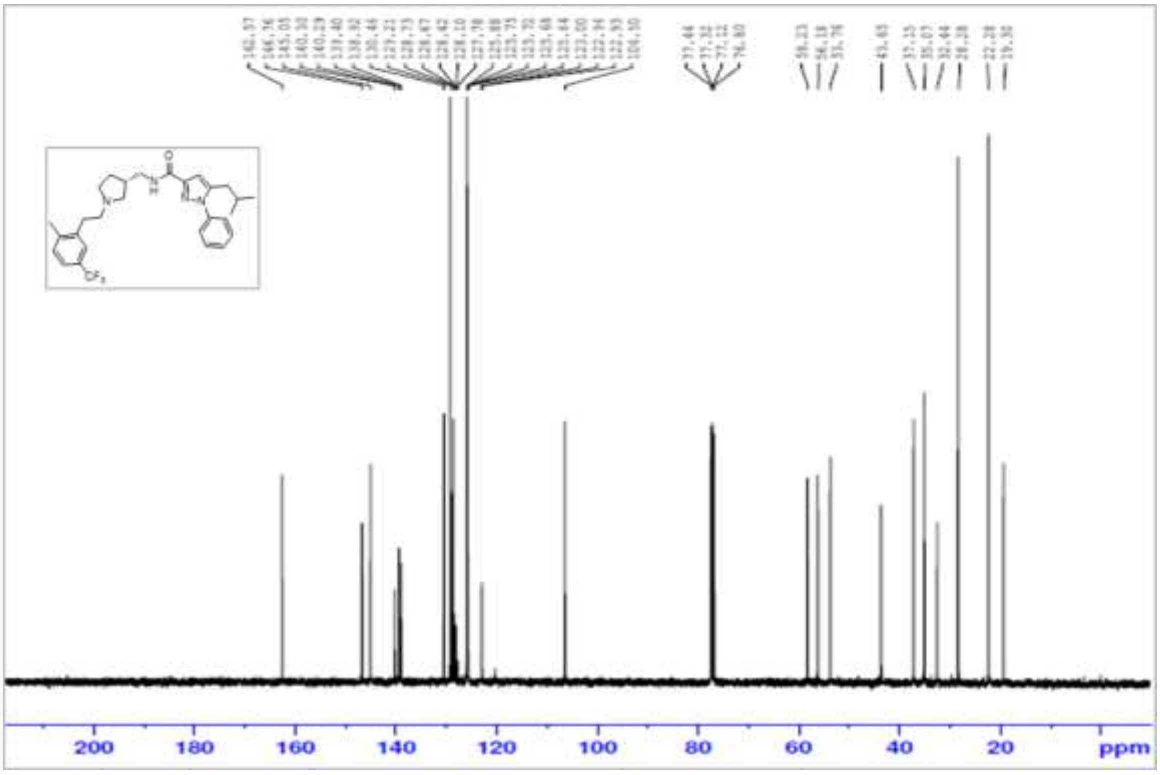

^{1}H NMR spectrum of (*R*)-5-Isobutyl-*N*-((1-(5-methyl-2-(trifluoromethyl)phenethyl)pyrrolidin-3-yl)methyl)-1-phenyl-1*H*-pyrazole-3-carboxamide (20p)

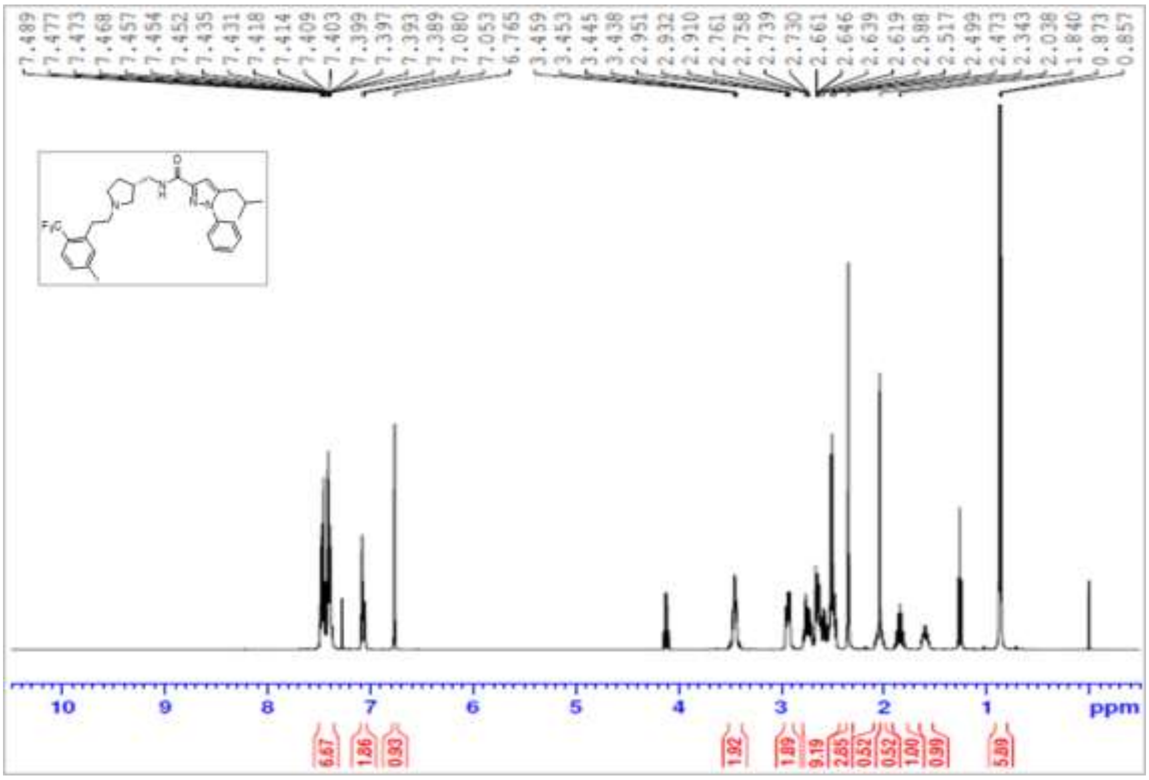

^{13}C NMR spectrum of (*R*)-5-Isobutyl-*N*-((1-(5-methyl-2-(trifluoromethyl)phenethyl)pyrrolidin-3-yl)methyl)-1-phenyl-1*H*-pyrazole-3-carboxamide (20p)

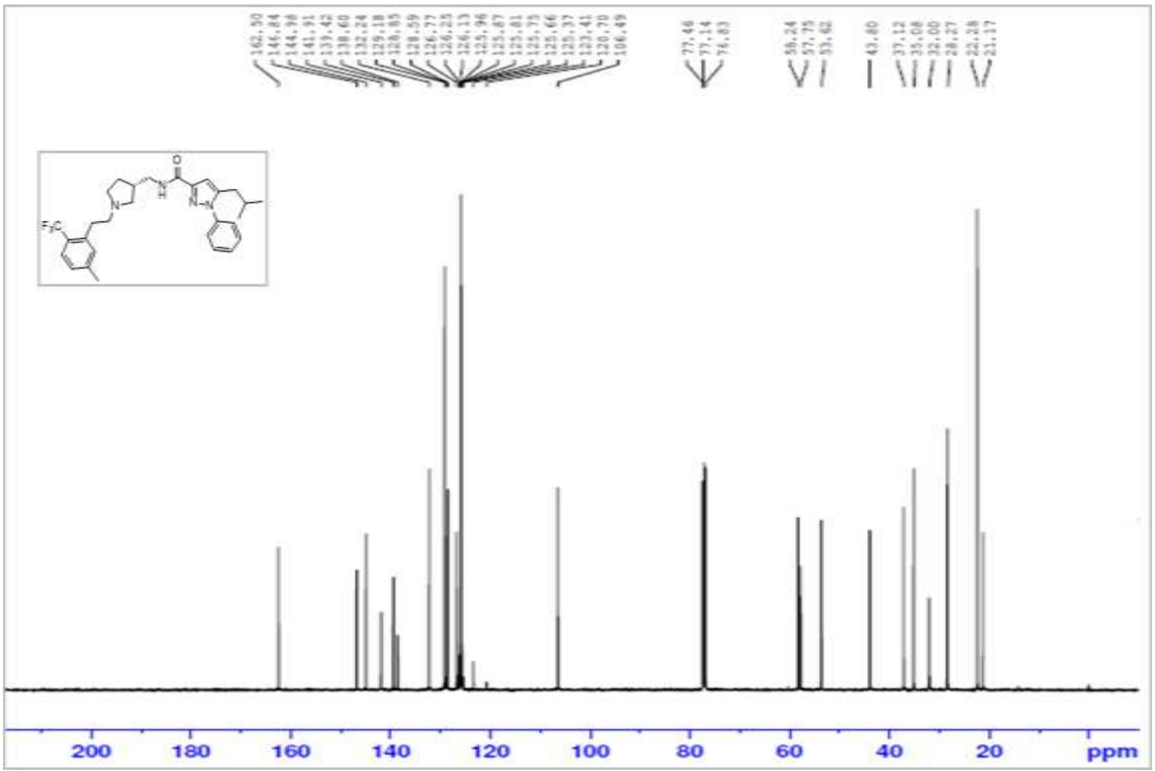

3. Pharmacophore Mapping

3.1. Methods

Using previously reported reference compounds[1], we developed a pharmacophore model. We put 2 in principal number for compounds of IC_{50} less than 1 μM and put 0 in maximum omitting features. For compounds of IC_{50} greater than 1 μM, principal number was set as 1 and maximum omitting features were set as 1. Eight reference compounds were used to generate a common feature pharmacophore model using HipHop algorithm implemented in Discovery Studio Client 2018. HipHop algorithm establishes qualitative common feature pharmacophore models without the use of activity data. The 3D arrangement of the currently developed pharmacophore features is slightly different from the previously reported pharmacophore because of different versions of the software.

3.2. Pharmacophore mapping

Ten ligand-based common feature pharmacophore models were generated by HipHop algorithm. Statistical results of the developed pharmacophore models are given in the supplementary table 1. The pharmacophore models are consisted of hydrogen bond acceptor, hydrophobic, and positive ionizable features. Entire pharmacophore scores range between 106.300 and 102.249. Higher ranking score implicates that the compound is less likely to fit the hypothesis by a chance correlation. We selected hypothesis 2 (Supplementary Table 1) for the further discussion and alignment of the **20n**. The hypothesis 2 is consisted of one hydrogen bond acceptor, three hydrophobic, and one positive ionizable features with the best fit value. Activities and fit values of reference compounds are shown in the supplementary table 2. The fit value of **20n** mapping is 2.57, which is well aligned with the activity (IC_{50} = 3.36 μM).

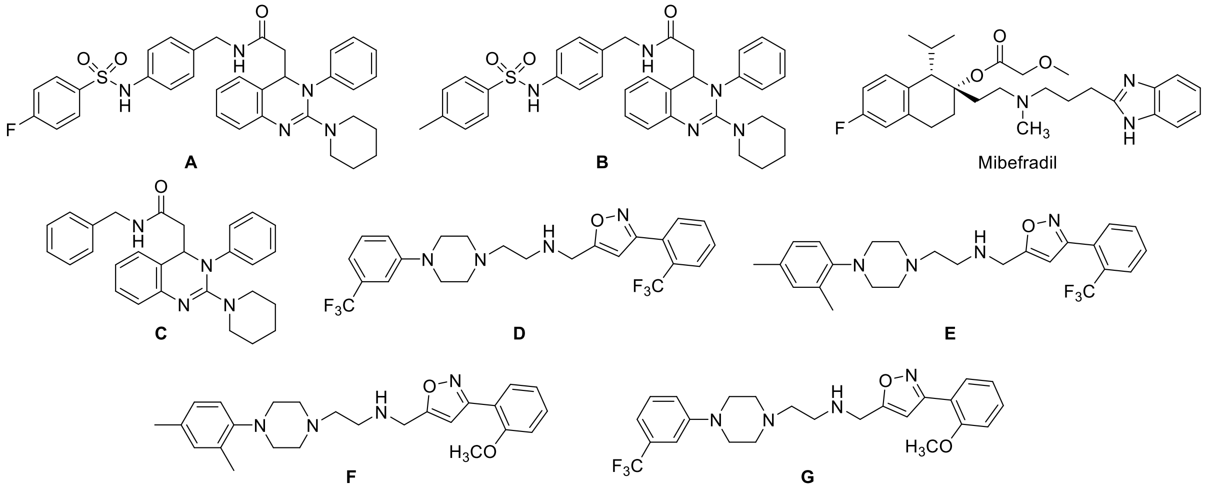

Supplementary Figure 1. Chemical structures of the reference compounds used to develop common feature pharmacophore.

Supplementary Table 1. Statistical results of a pharmacophore model generation.

No.	Features	Ranking Score	Direct Hit	Partial Hit	Max Fit
01	PHHHHA	106.300	11111111	00000000	6
02	PHHHA	104.929	11111111	00000000	5
03	PHHHA	104.881	11111111	00000000	5
04	PHHHHA	104.381	11111111	00000000	6
05	PHHHA	103.952	11111111	00000000	5
06	PHHHHA	103.519	11111111	00000000	6
07	PHHHA	103.496	11111111	00000000	5
08	PHHHHA	103.201	11111111	00000000	6
09	PHHHHA	102.457	11111111	00000000	6
10	PHHHA	102.249	11111111	00000000	5

P; Positive ionizable, H; Hydrophobic, A; Hydrogen bond acceptor.

Supplementary Table 2. Fit values of reference compounds and **20n**.

**Compounds**	**IC_{50} (μM)**	**Fit values**
**A**	0.2	5
**B**	0.25	3.63
**Mibefradil**	0.84	2.64
**C**	0.9	0.93
**D**	1.02	1.81
**E**	1.53	2.57
**F**	2.02	2.05
**G**	2.04	2.11
**20n**	3.36	2.57

4. Reference

1. Doddareddy MR, Jung HK, Lee JY, Lee YS, Cho YS, Koh HY, Pae AN. First pharmacophoric hypothesis for T-type calcium channel blockers. Bioorg Med Chem 2004;12: 1605-1611.
